# Pan-phylum Comparison of Nematode Metabolic Potential

**DOI:** 10.1371/journal.pntd.0003788

**Published:** 2015-05-22

**Authors:** Rahul Tyagi, Bruce A. Rosa, Warren G. Lewis, Makedonka Mitreva

**Affiliations:** 1 The Genome Institute, Washington University School of Medicine, St. Louis, Missouri, United States of America; 2 Division of Infectious Disease, Department of Internal Medicine, Washington University School of Medicine, St. Louis, Missouri, United States of America; 3 Department of Genetics, Washington University School of Medicine, St. Louis, Missouri, United States of America; McGill University, CANADA

## Abstract

Nematodes are among the most important causative pathogens of neglected tropical diseases. The increased availability of genomic and transcriptomic data for many understudied nematode species provides a great opportunity to investigate different aspects of their biology. Increasingly, metabolic potential of pathogens is recognized as a critical determinant governing their development, growth and pathogenicity. Comparing metabolic potential among species with distinct trophic ecologies can provide insights on overall biology or molecular adaptations. Furthermore, ascertaining gene expression at pathway level can help in understanding metabolic dynamics over development. Comparison of biochemical pathways (or subpathways, i.e. pathway modules) among related species can also retrospectively indicate potential mistakes in gene-calling and functional annotation. We show with numerous illustrative case studies that comparisons at the level of pathway modules have the potential to uncover biological insights while remaining computationally tractable. Here, we reconstruct and compare metabolic modules found in the deduced proteomes of 13 nematodes and 10 non-nematode species (including hosts of the parasitic nematode species). We observed that the metabolic potential is, in general, concomitant with phylogenetic and/or ecological similarity. Varied metabolic strategies are required among the nematodes, with only 8 out of 51 pathway modules being completely conserved. Enzyme comparison based on topology of metabolic modules uncovered diversification between parasite and host that can potentially guide therapeutic intervention. Gene expression data from 4 nematode species were used to study metabolic dynamics over their life cycles. We report unexpected differential metabolism between immature and mature microfilariae of the human filarial parasite *Brugia malayi*. A set of genes potentially important for parasitism is also reported, based on an analysis of gene expression in *C*. *elegans* and the human hookworm *Necator americanus*. We illustrate how analyzing and comparing metabolism at the level of pathway modules can improve existing knowledge of nematode metabolic potential and can provide parasitism related insights. Our reconstruction and comparison of nematode metabolic pathways at a pan-phylum and inter-phylum level enabled determination of phylogenetic restrictions and differential expression of pathways. A visualization of our results is available at http://nematode.net and the program for identification of module completeness (modDFS) is freely available at SourceForge. The methods reported will help biologists to predict biochemical potential of any organism with available deduced proteome, to direct experiments and test hypotheses.

## Author Summary

We reconstructed metabolic pathways of 23 organisms including 13 nematode species, using their complete deduced protein coding sequences and compared them to 10 non-nematodes. We observed that metabolic potential availability is concomitant with phylogenetic and/or ecological similarity, with the exceptions providing interesting case studies. We also studied changes in metabolic profiles under different developmental stages of 4 nematode species using stage-specific transcriptomic data. A comparison of the variation patterns in these profiles led to recognition of modules that share metabolic profiles at various life-cycle stages or during development. The undertaken analysis improved genome annotation and the obtained results provided insight into parasitism, resulting in identification of taxonomically-restricted pathways and enzymes that may provide new mechanisms for control of nematode infections.

## Introduction

The phylum Nematoda is one of the most diverse phyla among animals (with some estimates of the number of existing species being as high as 10 million [1]). The phylum contains a range of species occupying very different niches; including human parasitic species. Parasitic nematodes of humans are among the most important causative agents of neglected tropical diseases, with the morbidity from parasitic nematodes rivaling diabetes and lung cancer in disability-adjusted life years [DALY] measurements [2]). The WHO estimates that 2.9 billion people are infected with parasitic nematodes [3], making them the most common infectious agents of humans, especially in tropical regions of Africa, Asia and the Americas. The most common infections include 120 million cases for filariasis, more than 700 million each for hookworm infections and trichuriasis, and more than 1.2 billion for Ascaris [4]. While these numbers are ominous, large scale control and eradication programs have been largely successful for dracunculiasis (caused by *Dracunculus medenisis* [5]), and they are very promising for lymphatic filariasis (*Brugia* spp. and *Wuchereria bancrofti*) [6–8] and onchocerciasis (*Onchocerca volvulus*) [9]. However, elimination using existing approaches may be challenging for important helminthic infections such as soil-transmitted helminthiases due to the high risk of reinfection [10]. The dependence of control programs on a very limited number of drugs makes mass treatment programs vulnerable to evolution of drug resistance [11,12], as suggested by increased treatment failure rates observed in some areas [13–15]. Due to massive drug administration programs and improved hygienic practices, the 1.04 infections / person observed in 1930 has decreased to 0.606 infections per person in 2012 [4]. Moreover, the loss due to nematode parasites of domesticated animals and crops is estimated to be tens of billions of dollars per year [16,17]. Increasingly, development of resistance to anthelmintic drugs in veterinary medicine is very pronounced [18] especially since the employment of mass drug administration. While plant parasitic nematodes have devastating effects on crops costing $78 billion per year globally [17], using currently available nematicides to alleviate this burden is not possible because they are not environmentally safe. Hence, there is a pressing need to develop new anthelmintic treatments and pesticides [19] that are environmentally safe and efficient. Efforts for improving control have focused on identifying targets for drugs, vaccines and diagnostics. Speed and efficiency of such drug target identification will benefit from new insights into parasitic biological mechanisms.

Metabolic potential is one of the crucial factors that govern a pathogen’s development and pathogenicity [20–23]. In order to determine contrasts in the metabolic potential of parasites and non-parasitic organisms, we reconstructed the metabolic network in parasitic species and studied how they differ from non-parasitic sister species. Parasites may have significant evolutionary constraints for certain metabolic processes compared to their non-parasitic cousins (e.g. xenobiotic and toxic compound catabolism and transport [24,25]). On the other hand, they could allow for relaxed metabolic constraints in other processes (e.g. biosynthesis of important metabolites available from their host). In addition, many parasitic nematodes spend part of their life cycle outside the host (or have multiple hosts). This results in the evolutionary need to maintain or expand biochemical functions [26] in order to meet the requirements of several diverse developmental stages. Such comparisons of metabolic potential have been reported previously for individual parasitic nematodes [27–30], but a phylum-wide comparison has only recently become possible with many genomes available to cover the clades of the phylum as well as the many different niches and habits of parasitic worms. Emerging high-quality genomic databases for parasitic nematodes [31–33] now parallel the advances in publicly available data of other phyla. This should allow for the recognition of broader and more fundamental biological insights, through a transition from inter-species to pan-phylum analyses (e.g. [34,35] in other phyla).

The first global analysis of metabolic pathways in Nematoda used partial transcriptomes of 28 species and compared the extent of metabolic pathway representation [36–38]. A general congruence between enzymes associated with the major clades was observed, indicating that many pathways are conserved (with some relatively minor differences) within the nematodes, despite their diversity. These initial findings suggested that there are taxonomically restricted biochemical pathways and that they may serve to direct drug target definition. However, such conclusions were only suggestive and couldn’t be strongly supported from the available data because for most pathways the number of enzymes associated with each major clade correlated with the number of sequences generated. This suggested that the enzyme annotations were likely to be significantly incomplete. Since then, several studies compared metabolic pathways at a single-species level [39–41]. Also, the genome of the food-borne zoonotic parasitic nematode *Trichinella spiralis* (an extant member of a clade that diverged early in the evolution of the phylum) was recently sequenced [28], allowing pan-phylum comparison based on 4 species spanning the phylum Nematoda. However the inter-specific and pan-phylum studies undertaken thus far have not taken into consideration the topology of the pathways but only the individual enzymes.

Here, we perform a comparative metabolic analysis of 13 nematode species (including 5 non-parasitic and 8 parasitic species spanning the phylum [42]) and 10 non-nematodes, including hosts (S1 Table). The genomes for these species are in various stages of completion, with some still awaiting high-quality annotation. The comparison was performed at the metabolic module level rather than full metabolic pathways. As compared to full pathways, modules are smaller, more compact reaction cascades whose presence/absence can provide metabolic insight with higher resolution of detail (S1A Fig). This technique recognizes distinct mechanisms for the same overall metabolic pathway in different species. We analyzed over 500,000 proteins originating from the 23 species and compared the 127,000 predicted enzyme-encoding genes that were associated with over 10,000 KEGG Orthologous (KO) groups. Our modDFS program (publicly available at SourceForge and Nematode.net) helps interested users to use information from the KEGG database [38] and annotated proteomes to find potentially interesting differences between organisms that can then be studied in detail. To illustrate this, we performed *in silico* comparative metabolomics and report patterns of phylogenetic restriction of metabolic modules and examples of module diversification that can be used in obtaining better approaches to develop novel therapeutics. Finally, while genome annotations can be used to study the potential availability of various pathways to an organism, it doesn’t uncover any information about specific metabolic differences in the same organism under different conditions e.g. developmental stages. We therefore analyzed gene expression at different life cycle stages for 4 nematode species, including non-parasites and parasites.

## Results and Discussion

**Fig 1:**
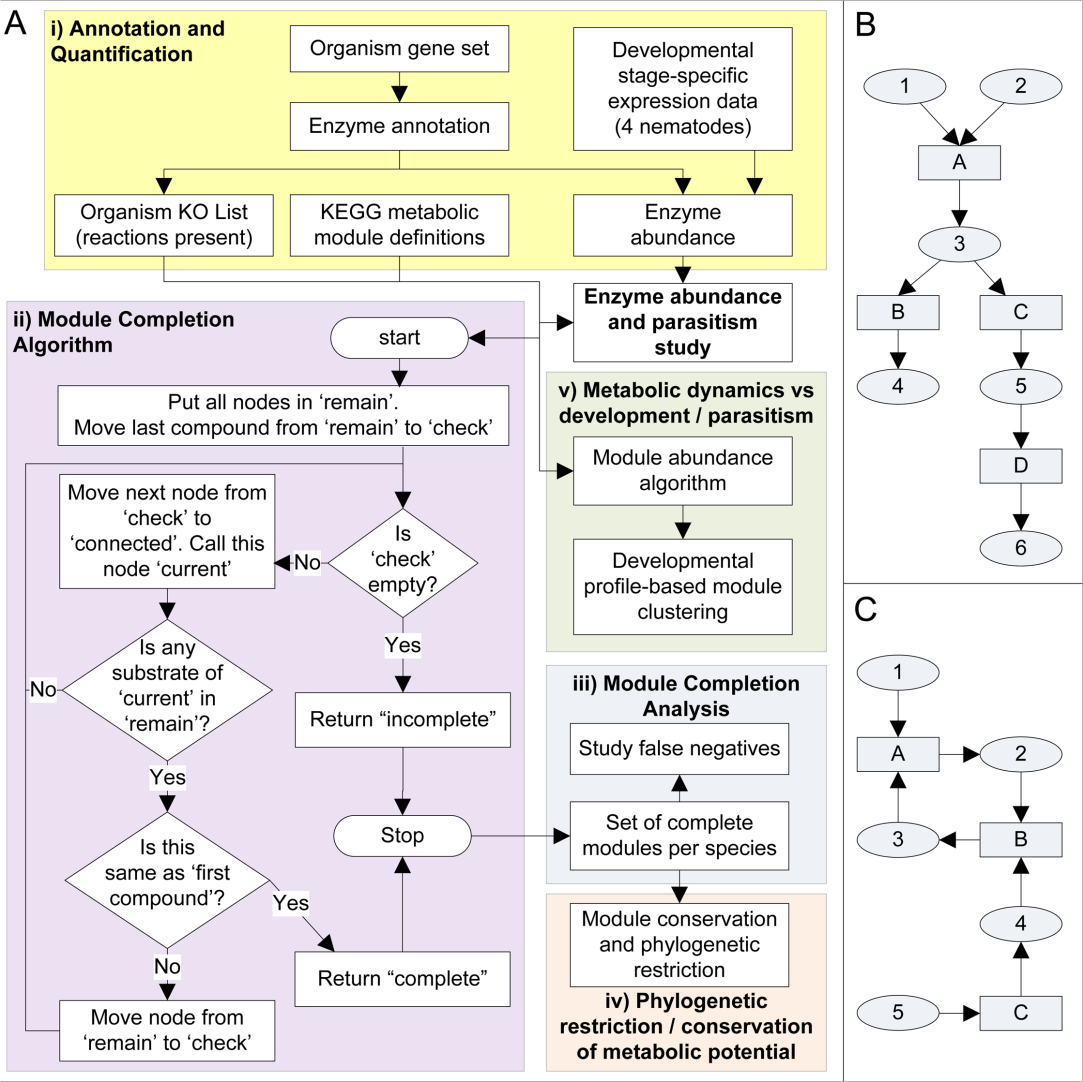
Analysis outline and module structure. A. The program logic flow chart for module completion algorithm is included. Data for module analysis is obtained by “Annotation and Quantification" (yellow box) to obtain data for module analysis. The 3 sections of analysis are illustrated with different colored boxes—Module completion; phylogenetic restriction / conservation of metabolic potential; Metabolic dynamics *vs*. development and parasitism. More detailed description of annotation, module completion and abundance algorithms are in the Methods section. B. Modules not containing a cycle can exhibit features such as multiple inputs (1 and 2) and forked network leading to multiple outputs (4 and 6). In general, more than 1 compound might be substrate-only (1 and 2 here), and hence needs to be treated as a ‘given’ compound for module completion determination. Rectangles are enzymes. Ovals are substrates/products. C. Network Reduction for simplifying completion algorithm. In this case with no reversible reactions, if none of the KOs representing reaction C is present in the organism, this leads to the following sequence of inferences. Reaction C absent = > no reaction has compound 4 as its product = > compound 4 is absent = > reaction B does not have 1 of its substrates = > compound 3 is absent = > reaction A does not have 1 of its substrates = > compound 2 is absent. This leaves the original network highly simplified and reduces it to just two nodes 1 and 5 (i.e. the original ‘given’ compounds) without any node connecting them.

Fig 1A presents the overall analysis approach, including the 5 major steps: i) annotation of metabolic enzymes (KO groups), ii) development of an approach to ascertain organism-specific pathway module completion, iii) pathway reconstruction for all nematode species and several non-nematode representatives with available genomic data, iv) *in silico* intra- and inter-specific comparative metabolomics and v) identification of developmental (i.e. condition-specific) pathway modules using transcription data of genes annotated with KOs. We developed modDFS (“completion of KEGG modules using Depth First Search”), an algorithm that determines whether a module is completely present within an organism. The requisite information to run modDFS is, a) enzyme content encoded in the deduced proteome (as KO groups; *see* Methods); and b) the KEGG database or metabolic module definitions. Canonical KEGG pathway maps provided the initial blueprint on which KO based enzyme assignments were overlaid to reconstruct each species metabolic pathways. The modDFS algorithm (outlined in Fig 1A, for details *see* Methods) is available for public use at http://sourceforge.net/projects/moddfs/ and http://nematode.net/Pathway_Modules.html.

### Reconstruction of metabolic modules enables accurate detection of complete modules

KEGG database provides gene-enzyme-pathway associations for many species, where such associations have validating experimental data available. However, for several parasitic nematodes the only feasible way to infer such information at present is through reliable computational prediction of enzyme encoding genes and their associations with pathway reactions and pathways. This is because many parasitic nematode genomes have only recently been published [28,43–46] and systematic enzyme survey studies for them are not feasible yet. Moreover, the graphical visualization of enzyme presence in pathways and modules available from KEGG is only based on single species data, unlike other KEGG-based data visualization methods [47]. We used KAAS for inferring enzymatic activities for the proteomes and in-house scripting for comparative visualization of that data overlaid on pathways (*see* Methods).

**Fig 2:**
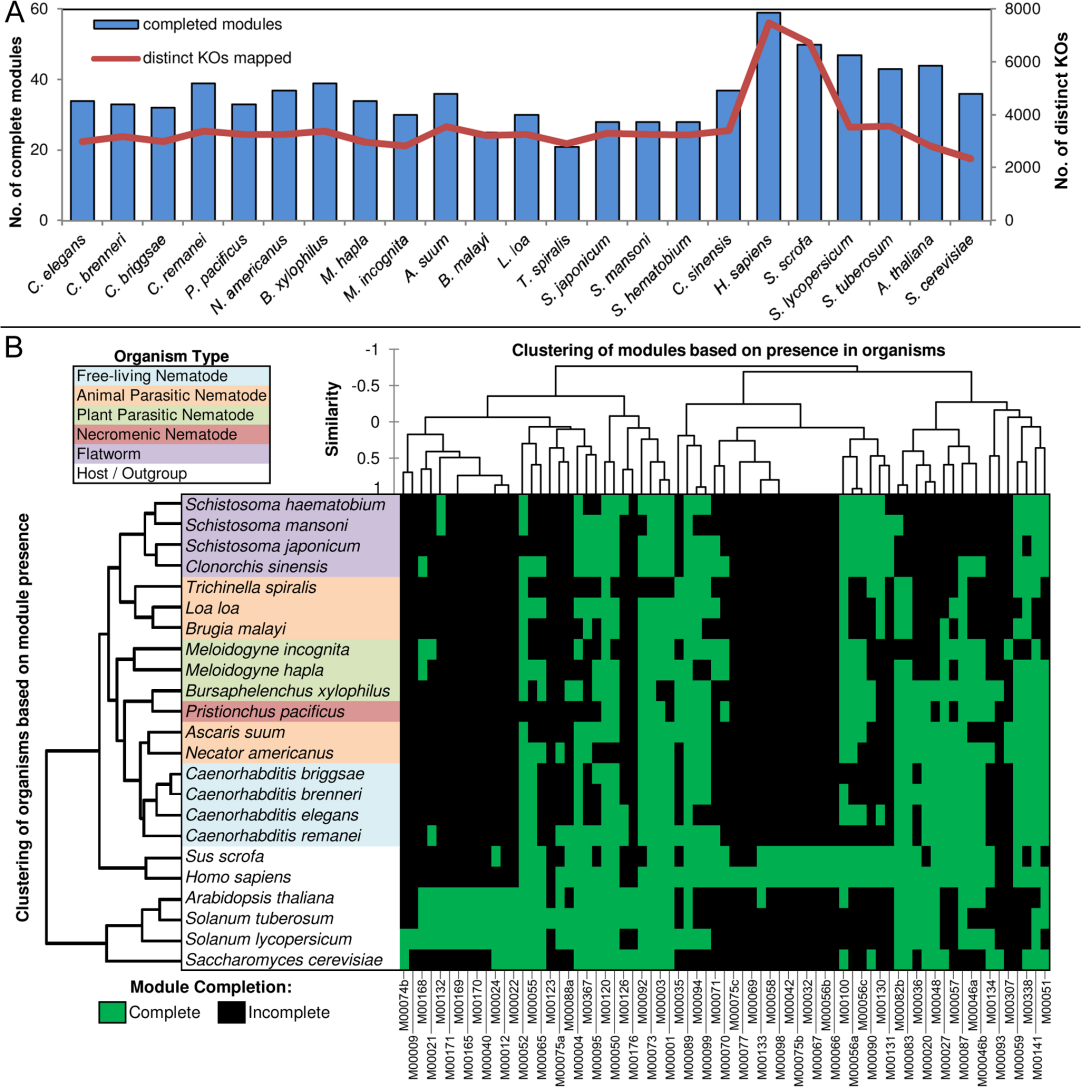
Enzyme annotation, module completion and phylogenetic distribution of metabolic potential. A. Number of complete modules and distinct KOs mapped to them. B. Clustering based on module presence correlation. A version of the figure that includes the module names is presented as S5 Fig.

Out of the 202 pathway modules present in the KEGG database, 92 were considered to be relevant (*see* Methods) and analyzed for the 23 species studied, including 14 forks from 6 forked modules (Fig 1B), 11 cyclic modules (Fig 1C), and 75 linear modules without cycles and forks (S2 Table; a visual representation of all reconstructed modules can be seen online http://www.nematode.net/Pathway_Modules.html). The reconstructed modules were classified into 2 tiers. Tier 1 represents ‘strictly complete’ modules (with at least 1 path from module’s beginning to end having all enzymes annotated in the deduced proteome of the species). While this requirement is needed for a reliable comparison of pathways among species, there are 2 main reasons that it may lead to false negatives: i) the draft nature of the genomes (especially non-*Caenorhabditis* nematode genomes) and ii) sequence diversification, which could lead to undetectable similarity at a primary sequence level. Hence, tier 2 modules were identified to capture false negatives as a result of missing genes. These represent modules that were ‘leniently complete’ (with at most 1 missing KO, such that, had it been detected it would have resulted in the module being tier 1). This resulted in reducing false negatives and increasing the number of complete modules (S2A Fig). Analysis of tier 1 modules showed that the worms (i.e. the roundworms phyla Nematoda and the flatworms phyla Platyhelminthes) had fewer metabolic modules available to them (average of 32) than the plants and animals analyzed (average of 49), which corresponded with the number of KOs associated with the respective proteomes (Fig 2A). This lower functional diversity relates to the less complex biology of the worm species included in our analysis, but in part may also arise from the draft nature of the genome and imperfect reaction annotation. The latter was confirmed by comparing the increase in completion rates resulting from allowing lenient completion. The % strict completion rates (i.e. the fraction of tier 1 modules) for non-nematode organisms tended to be higher (more than ~70, between 69 and 88, S2A Fig) than the worms (less than ~70, between 54 and 73). This is in part due to the selected host organisms having relatively well-studied and complete genomes.

#### Lenient completion can help improve gene predictions

An example of a ‘leniently complete’ module (S2B Fig) is illustrated with the completion of reaction steps for module M00050 (Guanine ribonucleotide biosynthesis) in the 13 nematode species studied. Under the strict completion definition, this module is not available to 3 nematodes (*Necator americanus*, *Meloidogyne incognita* and *T*. *spiralis*). While this may mean that 1 or more of these species really do not need this module in order to survive, a detailed analysis (S2B Fig) indicates that all these species are missing this module due to the lack of a single KO in their proteome (K00942—guanylate kinase—for the animal parasites *N*. *americanus* and *T*. *spiralis*; K01951—GMP synthase—for plant parasite *M*. *incognita*), with 3 out of 4 reaction steps being available to them. Since this KO is present in 10 other nematodes, the sequences from these genes can be used to search for a homologous enzyme in the worm’s genome assembly directly, blind to the genome annotation that was available. To determine if this approach would yield improved genome annotation and subsequently pathway completion, we built a Hidden Markov Model (HMM) of the K00942 orthologs of the other 10 species and searched the *N*. *americanus* assembly [43] with the model. S2C Fig shows a candidate region of the assembly that is found with high confidence matches to the HMM from other nematodes. The region overlaps partially with an annotated gene. Interestingly, the matching region also spans a gap in the assembly, which may explain why the gene could not be correctly annotated via sequence comparison. The species with the most incomplete modules due to 1 missing KO was *T*. *spiralis*, which has only 14 modules with more than 3 reaction steps complete, but 18 other modules were incomplete due to one missing KO (from each module). This approach could be used to improve the draft annotations of currently available genomes.

#### Sequence diversity is partly responsible for false negatives

We next explored the second possible reason that could contribute to increased false negatives, *viz*. the corresponding gene may evolve quickly compared to other enzyme-encoding genes, resulting in undetectable similarity at a primary sequence level. Consequently, a module in which that reaction is indispensable for its completion will falsely be reported to be incomplete in that species. To test this hypothesis, we analyzed the distribution of Nematoda-wide percent identities among KOs of tier 2 modules (i.e. leniently complete). Such absent reactions (i.e. only those absent reactions that, if they were present, would complete the module) corresponded to 49 KOs across all the nematode species. The corresponding “present KOs” set (i.e. the set of all KOs present in these modules) consisted of 193 KOs. We compared the mean percent-identity of all homolog pairs of these 2 sets (S3 Fig) (for “absent reactions”, the homologs pairs are from the species that have that enzyme annotated). Based on a permutation test with 1000 resamplings, the “absent” set had significantly lower mean sequence identity than the “present set” (S3A Fig; P≈0.05), with the actual difference being 2.8%. The difference is especially pronounced for genes with high sequence diversity, as the “absent” set contains a higher proportion of KOs with low mean sequence identity (<40%) as compared to the “present” set (S3B Fig). This implies that at least some of the missing enzymes are likely to be due to their fast-evolving nature, although this factor is unlikely to affect the recognition of majority of reaction activities in these species’ metabolic module completion using proteome data.

### Inter- and intra-phylum comparison of metabolic modules reflects phylogenetic and trophic relationships

#### Inter-phylum metabolic module examination

All of the modules that were found to be complete in at least 1 of the 23 studied species (77 modules out of the 92 analyzed) were used to study the conservation and diversification among the species based on their metabolic potential. While the modules representing core metabolic pathways are expected to be conserved over a broad phylogeny, other restricted modules are expected to be conserved among species that are phylogenetically close to each other. Any modules that defy this expectation are potentially important indicators of evolutionary adaptation related to the organism’s lifestyle niche. Based on the phylogenetic distribution of the pathway modules, we examined whether phylogenetically related species share common metabolic pathways, and whether there is a correlation between the metabolic physiology of these species and their trophic ecology.

The correlation coefficient for species that are phylogenetically close to each other (S4 Fig) tend to be higher compared to those which are more distant (e.g. average P = 0.80 among *Caenorhabditis spp*. vs. 0.59 for *Caenorhabditis spp*. to *Pristionchus pacificus*). Clustering based on presence of complete modules (Fig 2B) showed overall dendrogram topology largely following the phylogenetic relationship among the species, especially at the phylum level, with nematodes, platyhelminthes and other outgroups being more distant from each other compared to species within those groups (with the exception of 1 parasitic nematode branch clustering with flatworms rather than other nematodes; Figs 2B and S5). Platyhelminth clustering showed *Schistosoma haematobium* to be metabolically closer to *S*. *mansoni* as compared to other platyhelminthes, as expected from genome-wide comparisons [48]. Comparing these results to those based on just the KO content of these modules (i.e. blind to the module topology) demonstrates the expected improvement resulting from topology-based analysis. For example, ignoring topology information leads to some parasitic nematodes to be clustered with human and pig, rather than other worms (S6A Fig). These include the plant parasite *M*. *hapla* whose phylogenetic and ecological closeness with *M*. *incognita*, another plant parasite from the same genus, isn’t recognized based only on enzyme content. Clustering of the species based on the tier 2 modules (S6B Fig) led us to conclude that the tier 1 modules are more suited to ascertaining relationship to specific trophic ecologies, while the tier 2 modules are more appropriate for ascertaining absence of a module with high confidence.

A total of 77 metabolic modules are distributed across the 4 major species groups (non-parasitic nematodes; parasitic nematodes; flatworms; other outgroups/hosts) (S7A Fig). Thirty-five modules are represented in members of all 4 taxonomic groups, although only 6 of these are present in all 23 species (S7B Fig). These 6 are the most conserved modules representing core functions and include M00005 (PRPP biosynthesis from ribose 5P)—pentose phosphate pathway, M00049 (Adenine ribonucleotide biosynthesis)—purine metabolism, the (GlcNAc)2-(Man)5-(Asn)1 branch of M00074 (high mannose type N-glycan biosynthesis)—N-glycan biosynthesis pathway, the malonyl-[acp] branch of M00082 (Fatty acid biosynthesis, initiation module)—fatty acid biosynthesis pathway, M00086 (beta oxidation, acyl-coA synthesis)—fatty acid metabolism pathway, and M00118 (Glutathione biosynthesis from Glutamate)—glutathione metabolism pathway. Furthermore, there are 3 modules that are present in all but 1 species (n = 22). These are module M00120 (Coenzyme-A biosynthesis from pantothenate)—Pantothenate and CoA biosynthesis, absent in *N*. *americanus*; M00089 (triacylglycerol biosynthesis)—glycerolipid metabolism pathway, absent in *S*. *cerevisiae*; and M00073 (N-glycan precursor trimming)—N-glycan biosynthesis pathway, absent in *T*. *spiralis*, though the latter 2 are tier 2 modules (present under lenient definition). We note that *S*. *cerevisiae* is known to have an enzyme that provides the requisite activity for the missing reaction step, thereby synthesizing triacylglycerol [49]. Cases like this further justify our firm acceptance of detected modules but caution in interpreting tier 1 absence of a module.

There were modules which were conserved throughout phylum Nematoda but absent in at least 1 other taxonomic group or species. For example, M00087—beta-oxidation in fatty acid metabolism pathway. Beta-oxidation is a lipid metabolic pathway present in the plants and animals, but the full pathway is absent in 3 flatworm species (*S*. *mansoni*, *S*. *japonicum*, *S*. *haematobium*) and *S*. *cerevisiae*. Schistosomes are incapable of *de novo* synthesis of free fatty acids and must scavenge phospholipid and triacylglycerol precursors from the host [50]. In the *S*. *mansoni* genome, the genes necessary for a complete beta-oxidation pathway have been identified, and it has been speculated that this pathway may operate in reverse [51]. We note that while the 3 schistosomes are capable of initial beta-oxidation for synthesis of palmitoyl-coA (M00086), they lack the subsequent module for complete beta-oxidation (M00087). While flatworms lack *de novo* fatty acid biosynthesis, *S*. *mansoni* and some others are known to be capable of fatty acid chain elongation [50,52,53]. Our results show that elongation module M00083 is present in *S*. *mansoni* under tier 2 definition but absent from the other flatworms. The absence of most enzymes in this module among 4 different species of parasite (3 flatworms and the plant parasite *M*. *incognita*) may indicate that loss of these genes is related to parasitism. Specifically, these organisms have evolved mechanisms to either: a) circumvent the consequences of unavailability of long chain fatty acids, b) harvest long chain fatty acids from the host, or c) develop alternative mechanisms and activities for fatty acid elongation.

#### Intra-phylum Nematoda metabolic module examination

None of the modules studied is conserved in and exclusive to nematodes (i.e. present in all nematodes yet absent in all non-nematode species). A total of 51 modules were available to at least 1 of the nematodes studied (S2 Table), of which 40 were represented in at least 1 member of the 3 nematode groups (non-parasitic, animal-parasitic and plant-parasitic nematodes; S7C Fig), and only 8 of these are present in all 13 nematode species (S7D Fig).

Among the nematodes, most of the clustering is consistent with the taxonomic clades (phylogeny based on Blaxter et al. [42]) and lifestyle of the species. All non-parasitic *Caenorhabditis* species cluster together. *P*. *pacificus*, which is a necromenic species and hence has certain metabolic potential similarities with parasites [27], clustered with *Bursaphelenchus xylophilus*. *B*. *xylophilus* is a plant parasitic nematode that combines fungal feeding, plant parasitic and insect-associated stages [54]. It represents a recent, independent origin of plant parasitism in nematodes, ecologically and taxonomically distinct from other nematode plant parasitic species [45]. Interestingly, while *B*. *malayi* and *Loa loa—*both of which are filarial parasites—cluster together, the third clade III non-filarial animal parasite *Ascaris suum* clusters with clade V species. Our results indicate that the soil-transmitted *A*. *suum* is metabolically more closely related to the clade V soil-transmitted parasitic hookworm that also has non-parasitic stages (unlike the filarial parasites).

We next examined taxonomic restrictions of metabolic modules within Nematoda by comparing i) intra-genus module completions, using the *Caenorhabditis* as an example, since we had 4 species from this genus; ii) non-parasitic species from same clade, i.e. *Caenorhabditis spp*. vs. *P*. *pacificus* and iii) parasitic species in relation to the host.

i) Intra-genus comparison among *Caenorhabditis spp*. We compared the metabolic module completion in the 4 worms of the genus *Caenorhabditis* to find the core metabolome of this free-living genus. Forty-four out of 92 modules were present in at least 1 species (S8A Fig), out of which 29 were present in all 4 species (S8B Fig). Furthermore, 15 out of these 29 modules were not conserved throughout the 23 studied species even under tier 2 definition. This included modules for biosynthesis of fatty acids, nucleotides, glycans and the amino acids Serine and Cysteine (S2 Table). For the non-parasitic nematodes (as well as the non-parasitic stages of the parasitic nematodes) nutrients are limiting and full range of biochemical reactions are needed to synthesize metabolites *de novo*.

ii) Non-parasitic vs necromenic worms: impact of close association with “hosts”. We next compared the 4 *Caenorhabditis* species with *P*. *pacificus*¸ a related nematode in clade V which is non-parasitic but is associated with scarab beetles during one stage of its life cycle [27,55]. While 23 of the 29 core *Caenorhabditis* modules were also present in *P*. *pacificus* (S8C Fig), 3 modules were *P*. *pacificus* restricted: M00070, M00090 and M00134. The module for glycosphingolipid biosynthesis (lacto series; M00070, Glycosphingolipid biosynthesis—lacto and neolacto series pathway) had both its reaction steps absent in the *Caenorhabditis spp*. The modules for Phosphatidylcholine biosynthesis (M00090, Glycerophospholipid metabolism pathway) and polyamine biosynthesis (M00134, Arginine and proline metabolism pathway) were absent in the *Caenorhabditis* worms due to absence of 1 reaction step each. Close association of *P*. *pacificus* with other organisms during part of its life cycle has led to it potentially gaining novel genes by horizontal gene transfer [56]. A close examination of the blast search of these three enzymes against NCBI’s NR database revealed that the best matches of *P*. *pacificus* gene encoding the enzyme arginase (K01476) were primarily sequences from nematodes and bacteria sequence matches (S3 Table). The nematoda homologs had a sequence identity of *ca*. 60% (primarily clade V, IV, and III nematodes), followed by bacterial sequence identities of *ca*. 40%, indicating possible horizontal gene transfer of a bacterial arginase gene to Nematoda after the divergence of the branch leading to clades V&III. Association with other organisms also results in the worm losing certain genes that can be compensated by external availability of the associated metabolites during the necromenic stage of its life cycle. Loss of genes could also result from development of divergent functions and gene expression even among conserved stages (e.g. [57]). This is consistent with our observation of some modules absent in *P*. *pacificus* yet present in all the *Caenorhabditis* species (S8C Fig): M00048 (Inosine monophosphate biosynthesis—purine metabolism pathway) and M00055 (N-glycan precursor biosynthesis—N-glycan biosynthesis pathway). We examined in details the glycan-related pathways, to illustrate the strengths and weaknesses of our approach. There were 10 modules representing N- and O-Glycan biosynthesis pathways. N- and O-Glycans are major constituents of glycoproteins in eukaryotes, and they have attracted significant attention in parasitic nematodes due to their immunogenic and immunomodulatory nature [58]. The main interface between the parasitic nematodes and its host is the cuticle surface, which is covered by a carbohydrate-rich glycocalyx resulting in the protein glycan constituents of this cuticle surface coat being involved in host-parasite interaction. The glycosyltranferases and glycosidases required for the biosynthesis of the N-glycan core are generally conserved between mammals and helminths, leading to creation of the common core of the complex N-glycan [59]; this is consistent with the observed conservation of M00073 in our study although some enzymes of M00055 seem to have been lost in some parasitic species (S2 Table). Other modules involved in glycan biosynthesis were taxonomically restricted. Some experimentally-determined worm O-glycan modifications—glucosylation, glucuronidation, [59] and methylation [58]—were not explicitly detected in the present analysis based on KEGG modules indicating a need for analysis based on multiple databases or published reports. Furthermore, some nematodes display extensive fucosylation that may assist in parasitism, however, such information cannot be obtained through this analysis because they concern parts of the overall metabolic network not captured by the defined metabolic modules (e.g. S9 Fig). Hence, based on our analysis and literature support we were able to partially reconstruct glycan biosynthesis in parasitic helminthes and compare it to the mammalian complement. For instance, the complex N-glycans of mammals elaborate on the core structure quite differently from helminthes (S10 Fig). Mammals contain sialyltransferases that add sialic acid residues to their glycans, but helminth N-glycans are known not to contain sialic acid residues. This is consistent with our failure to annotate any sialyltransferases in any of the roundworms and flatworms that we studied, except the non-parasitic *C*. *brenneri* which has a protein annotated as sialyltransferase-6 (K00781). Since no sialyltransferase is detected in most helminths, even the helminth O-glycans aren’t expected to contain sialic acid residues. Indeed, O-glycan analyses of nematodes and flatworms show no evidence of sialylated glycans. Furthermore, non-sialylated β-galactose [60] and N-acetyl galactosamine [61] are well-known antigens found in parasitic roundworms and flatworms (S10 Fig). The unexpected presence of sialyltransferase in *C*. *brenneri* is potentially an interesting avenue of further study. Taxonomic restrictions were detected among the plants and plant parasitic nematodes too. It is known that glycosaminoglycans (GAGs) are absent in plants, but present in invertebrates [62]. Indeed, the plant parasites *B*. *xylophilus* and *M*. *incognita*, and *M*. *hapla* possess GAG biosynthesis module (M00057), as expected. Plants are also known to lack GalNAc-type O-glycosylation (M00056) [63], which is conserved among animals here, with a branch of the module being at least leniently complete in all animal species.

Overall, our analysis was able to detect pan-Phylum conserved and taxonomically restricted pathway modules. The results also indicate that integrating chemical analysis of glycan structures into future analyses of helminth genomes could give better prediction based on genomic analysis.

iii) Module diversification among pathogens and hosts provides potential for novel therapeutics. No modules were conserved in all parasitic nematodes but absent from all non-parasitic nematode species. This means that none of the metabolic modules studied here is highly parasitism specific without any role in non-parasites. Such lack of easily recognized parallels among all parasites is likely a result of the varied niches that these parasitic nematodes populate. For example, even among animal parasites there are substantial differences in lifestyle: hookworm attaches to the wall of the small intestine and feeds on the host's blood, whipworm attaches to the lining of the colon and cecum where they feed on tissue fluids, blood and mucosal epithelium, ascaris lives in the lumen of small intestine and feeds on the intestinal contents, and filaria feed on blood and lymphatic tissue and fluid. We therefore investigated the differences in metabolic processes between parasites and hosts which can contribute to our understanding of parasitism and drug target discovery [64]. There is a perception that parasites have lost functions through reductive evolution by relying on the metabolic capacity of their hosts. Notably, the clade I nematode *T*. *spiralis* and the clade III filarial nematode *B*. *malayi* have significantly fewer potential complete metabolic modules associated with their proteomes compared to other nematodes (p = 0.0006 and 0.04, respectively). This is partly due to draft nature of the genomes, but is also potentially a consequence of these being obligate parasites. Both of these parasites do not have a free-living stage in their entire life cycle and could consequently derive key metabolites and enzymes from their hosts and/or intermediate vectors, resulting in their clustering close to each other and separate from non-parasites and soil-transmitted helminth parasites as shown in Fig 2B. Furthermore, *B*. *malayi* also has endosymbiotic bacteria—*Wolbachia*—which are likely to provide metabolites and enzymes to the worm [65], for example, it has been proposed that the absence of pyrimidine metabolism in *B*. *malayi* (S2 Table) may be replaced with *de novo* pyrimidine synthesis of *Wolbachia* [66]. Many other parasitic nematodes spend part of their life cycle outside the hosts, and therefore may experience evolutionary pressure to maintain or expand biochemical functions required for their non-parasitic stages relative to obligate parasites [26].

Our approach examined the module structures to find cases of variation in enzyme usage between the parasite(s) and the corresponding host, for the pairs of human and human hookworm *N*. *americanus*, pig and pig roundworm *A*. *suum*, and also compared the plants (*A*. *thaliana*, *S*. *tuberosum* and *S*. *lycopersicum*) and plant parasites (*M*. *incognita*, *M*. *hapla* and *B*. *xylophilus*) in the dataset. We observed primarily 3 types of variations: i). host and parasite use partially overlapping set of KOs to complete modules, ii). host and parasite use exclusively non-homologous enzymes to complete the same reaction step, and iii). host and parasite use highly conserved enzymes, but a subset of these conserved proteins have nematode-specific features (e.g. indels—insertions or deletions) with potential functional and evolutionary relevance [67].

**Fig 3:**
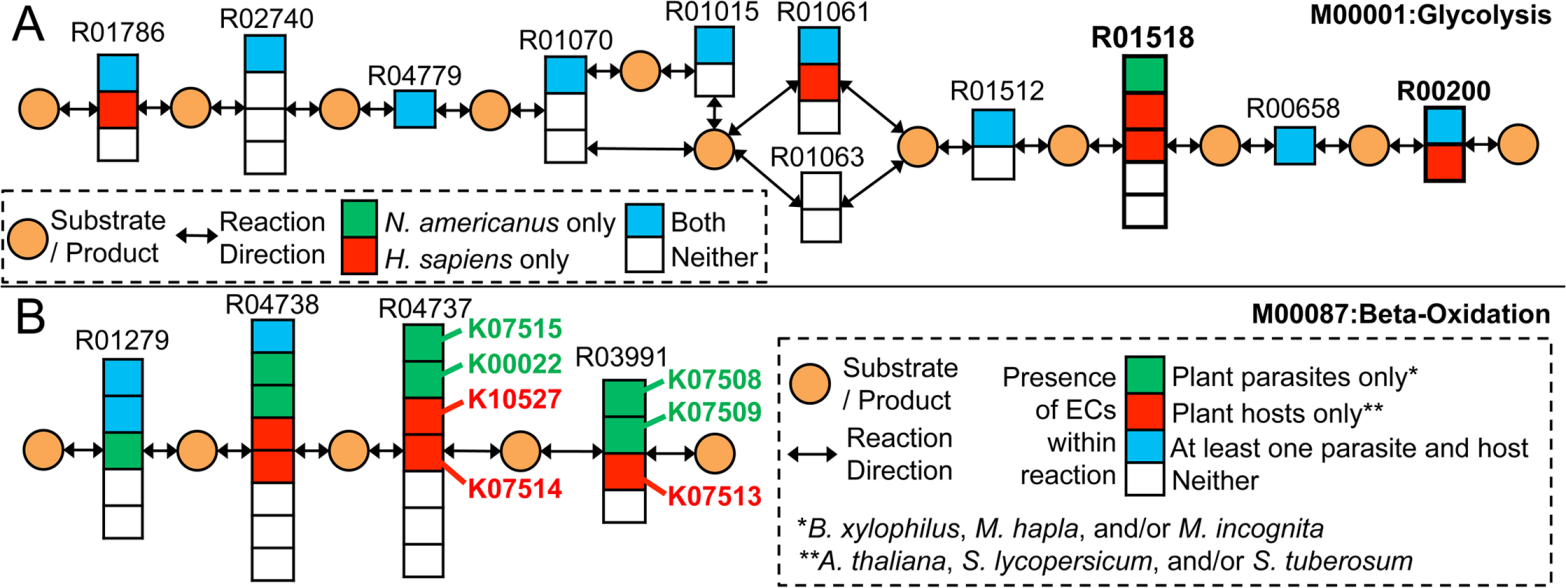
Enzyme diversification between host and parasite. A. Enzyme diversification between human and human hookworm *Necator americanus*. Reactions R01518 (2-Phospho-D-glycerate 2,3-phosphomutase) and R00200 (pyruvate kinase) are indispensable steps for the completion of M00001 (Glycolysis Embden-Meyerhof pathway). *N*. *americanus*, a human hookworm parasite, uses KO K15633 for R01518, which the human proteome lacks. Human proteome has genes mapping to K01837 and K01834 instead, which completes the same reaction. For R00200, both Human and *N*. *americanus* have K00873 available for completion, but only human have K12406 as an alternative for reaction completion. B. Enzyme diversification between plants and plant –parasitic nematodes. Plants have many more examples of exclusive KO usage between hosts and parasites. Module M00087 (beta-oxidation) is an interesting case where the last 2 reactions—R04727 and R03991—use different KOs for completion in all the 3 plants vs the 3 plant parasite nematodes. Different KOs are likely to be mapped to genes that are not orthologous to each other, providing an opportunity to target the parasite-specific activity.

The first type of variation is exemplified in Fig 3A, R00200 is a reaction catalyzed by pyruvate kinase, an enzyme shown to play an important role in anaerobic metabolism in *A*. *suum* [68]. As shown in the figure, the host (human) uses an additional KO, along with a KO that it shared with the parasite hookworm. It is possible that targeting the common KO in such cases can knock out the module entirely in the parasite with acceptable concomitant impact on host metabolism. For example, it has been reported that adult *A*. *suum* and heartworm may have adapted to their oxygen-poor environment by developing an anaerobic energy-generation system distinct from their mammalian host pathways, making it a promising target for the development of next-generation macrofilaricides [30]. On the other hand, when it is the parasite that has an additional KO (e.g. R04325 in module M00048 in S4 Table), if the additional KO is targeted then it renders the module completion inefficient only in the parasite. The second type of variation (distinct non-overlapping sets of KOs, Table 1) is exemplified in the reaction R01518 in Fig 3A. This reaction is catalyzed by phosphoglycerate mutase, which has been shown to have a unique form in nematodes compared to mammals, and has been suggested as a promising drug target in parasitic nematodes [69]. Here, we identified this, already reported, potential drug target by looking for non-homologous enzyme functions within metabolic modules. Targeting the parasite-specific enzyme can avoid adverse impact on the host, and strategic analysis of metabolic modules can provide insights into potential drug targets. As expected, higher number of non-overlapping KO reactions were identified when comparing the plant parasites with plants, as compared to the Human-*N*. *americanus* and Pig-*A*. *suum* pairs. Module M00087 (beta oxidation) is especially notable with mutually exclusive use of enzymes by all plant parasite-host pairs; Fig 3B). We note that the suggestion here isn’t that all plant parasite worms are suitable pairs for all plants (e.g. *B*. *xylophilus* isn’t known to infect any of the plants in our dataset), but that broadly conserved differences in module completion between plants and parasite worms can guide therapeutic studies. Finally, the third type of variation included nematode-specific indels in highly conserved proteins that warrant further studies due to the indel-based targeting potential [70]. We identified that >40% of the enzymes common between *N*. *americanus* and human had taxonomically restricted indels (S11 Fig and S5 Table). Insertions could create additional loops in the nematode proteins that may introduce novel functional characteristics specific to Nematoda, and may suggest diversification of existing protein folds during nematode evolution. Systematic analyses to identify such nematode-specific indels have been performed and functional relationship inferred [67].

**Table 1:** Examples of exclusive enzyme usage by parasite and host.

Module id	Module descriptor	Reaction Step	Parasite—Host pair
M00001	Glycolysis (Embden-Meyerhof pathway), glucose = >pyruvate	R01518*	*H*. *sapiens—N*. *americanus*; *S*. *scrofa—A*. *suum*
M00003	Gluconeogenesis, oxaloacetate = > fructose-6P	R01518*	*H*. *sapiens—N*. *americanus*; *S*. *scrofa—A*. *suum*
M00087	beta-Oxidation	R03991+	all plant parasites—all plants
M00087	beta-Oxidation	R04737+	all plant parasites—all plants
M00087	beta-Oxidation	R04738+	*M*. *hapla*—all plants; *M*. *incognita—*all plants; *B*. *xylophilus*—*A*. *thaliana*
M00089	Triacylglycerol biosynthesis	R02241+	*M*. *hapla*—all plants; *M*. *incognita—A*. *thaliana*
M00118	Glutathione biosynthesis, glutamate = > glutathione	R00894+	all plant parasites—*S*. *lycopersicum*; all plant parasites—*A*. *thaliana*
M00082a	Fatty acid biosynthesis, initiation, malonyl-acp	R01624+	*M*. *hapla*—all plants; B. xylophilus—*A*. *thaliana*
M00082a	Fatty acid biosynthesis, initiation, acetoacetyl-acp	R04355+	*M*. *hapla*—all plants
M00120	Coenzyme A biosynthesis, pantothenate = > CoA	R00130+	*B*. *xylophilus*—all plants
M00120	Coenzyme A biosynthesis, pantothenate = > CoA	R03035+	all plant parasites—all plants

* R01518 is the only reaction that uses mutually exclusive KOs in the 2 animal-parasite pairs studied.

+ The plants have a lot more such cases and we list only the cases involved in the modules that are complete in all 3 plant parasites and all 3 plants in the dataset. The details of these comparisons can be obtained from S4 Table.

Our pathway completion and comparative metabolomics can also be combined with the identification of putative chokepoint enzymes (which catalyze reactions that either uniquely consume a specific substrate or uniquely produce a specific product) to identify potential targets for drug discovery. A recent paper identified and characterized metabolic chokepoint reactions and enzymes in nematodes [20]. Of the 477 ECs reported in that study, 103 are part of the 77 modules that are complete in at least 1 of our 23 species. These represent 35.4% of the 291 ECs that are part of our 77 complete metabolic modules.

### Gene expression dynamics during the course of development defines metabolic module clusters with conserved expression profiles

The reconstructed pathways provide an indication of the metabolic potential. However by themselves, they do not establish whether a complete pathway is operative in an organism under a particular developmental stage or condition. Such context-dependent metabolic potential can be studied by using large-scale proteomic and enzyme kinetics data. However, such data does not exist for parasitic nematodes. Therefore, we used RNA-seq based expression to examine the metabolic dynamics during development and investigate context-dependent metabolic features in the phylum Nematoda. The nearly complete transcriptional profiles obtained using RNA-seq data for 3 parasitic nematode species [43,71,72] and *C*. *elegans* [73] (S6 Table) were used to assign an abundance value to every complete metabolic module using an enzyme cascade bottleneck calculation (*see* Methods). As expected, the module abundances are found to be relatively dynamic over the course of organism development. Moreover, clustering of modules according to their abundance profiles across developmental stages uncovers interesting contrasts in development profiles of the sets of modules. We discuss the results for 1 non-parasitic (*C*. *elegans*) and 1 parasitic (*B*. *malayi*) species here (see S1 Text for *A*. *suum* and *N*. *americanus*).

**Fig 4:**
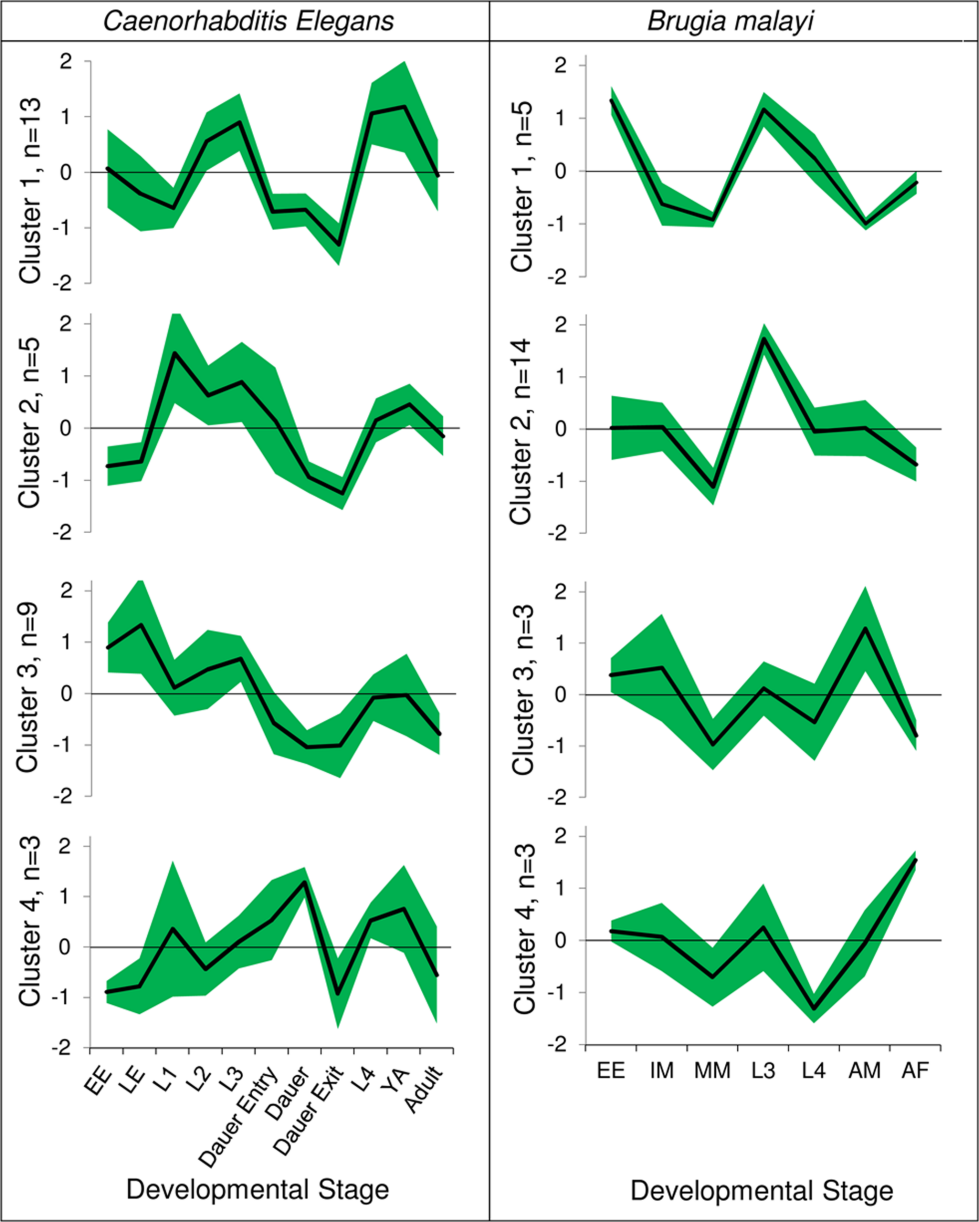
Developmental stage based metabolic profiles for *Caenorhabditis elegans* and *Brugia malayi*. The clusters are based on similarity between abundance profiles across developmental stages (*see* S12 Fig). The black line (green bounds) represents the mean (standard deviation) of the Z-score of abundances for that developmental stage of all modules in the cluster. The Z-score is a measure of relative over- of under-abundance of the module in that developmental stage compared to its mean over all stages. EE: Early Embryo; LE: Late Embryo; IM: Immature Microfilariae; MM: Mature Microfilariae; YA: Young Adult; AM: Adult Male; AF: Adult Female.

Of the 34 potentially complete modules in *C*. *elegans*, 30 have non-zero abundance in all developmental stages and 1 (M00004) has non-zero abundance in all except the L1 stage. Furthermore, 3 other modules (M00056a, M00056c and M00126) had 0 abundance in all stages due to no detectable expression of some critical enzyme in the module. For each of the 31 modules with detectable abundance levels, we determined the differentially expressed modules using the Z-score statistic (based on the mean and standard deviation of that module’s abundance across all the stages). The Z-scores help identify the modules that are relatively over or under abundant in any given stage as compared to that module’s mean abundance level across all the stages. These Z-scores were then used to obtain clusters of modules that have similar abundance profiles across the developmental stages (Fig 4). The adult and the dauer stage module abundances were generally low as compared to other stages with only a few modules being overabundant (7 modules with >0 Z-scores), and hence they cluster together (S12A Fig). In contrast, the young adult stage has consistently high abundance Z-scores (with 21 modules having >0 Z-scores). Four clusters of co-expressed modules were defined (plotting the abundance Z-scores against the ordered developmental stages, Fig 4). Two clusters included 22 of the 31 modules (clusters 1 and 3) that differ primarily in overabundance in later stages (L4 and Young Adult, overabundant in cluster 1 modules) vs. embryonic stages (overabundant in cluster 3). Cluster 3 includes biosynthesis modules for fatty acids, beta alanine, guanine nucleotides, cysteine and PE. Cluster 1 includes biosynthesis of serine, adenine nucleotides, UMP, glycosaminoglycans and N-glycans, PRPP and CoA along with modules for pentose phosphate pathway, sphingosine degradation and PI metabolism. Cluster 2 (overabundant in L1) includes modules for glycolysis, gluconeogenesis, acyl-CoA synthesis, glutathione biosynthesis and C1-unit interconversion. L1 longevity has been previously linked with metabolic rate and starvation [74]. Cluster 4 (considerably overabundant during the dauer stage, and underabundant during the L2 stage) includes modules for GABA shunt and biosynthesis of IMP, pyrimidine ribonucleotides and triacylglycerol, which is consistent with the known enhancement of cellular maintenance and detoxification processes in dauer stage [41].

Performing analyses of module abundances in different available developmental stages of the filarial parasitic nematode *B*. *malayi* defined 4 clusters, with uniformly low abundance of almost every metabolic module in the mature microfilariae as compared to other stages (S12B Fig). On the contrary, most modules (19 out of 25, Clusters 1 and 2) with only a few exceptions that partly define clusters 3 and 4, are overabundant in the L3 stage. The adult stages do not cluster with each other. The male have similar abundance pattern to immature microfilariae and the female have low abundances in a pattern similar to mature microfilariae (with 3 modules being notable exceptions). Fig 4 shows that a majority of modules (14 out of 25) share their module abundance profiles closely (cluster 2), with significant overabundance in the L3 stage. Cluster 1 (comprising modules for fatty acid biosynthesis and methionine and sphingosine degradation) is differentiated from these by a sharp fall in abundance from the eggs and embryonic stage to immature microfilariae stage. They also show relative underabundance in the adult male, as compared to all other clusters. Clusters 3 and 4 show a characteristic overabundance in adult stages (male for cluster 3, and female for cluster 4). Cluster 3 includes glycolysis, glutathione biosynthesis and CoA biosynthesis. Cluster 4 has low relative abundance before adulthood (L4 stage) and comprises modules for PI metabolism and biosynthesis of PRPP and C10-C20 isoprenoid. Previously it has been suggested that transcriptional differences between immature and mature microfilariae may be relatively small despite the change in infectivity [72]. However, we observed that a majority of complete metabolic modules have differential abundance between these 2 stages (14 out of 25 having a Z-score difference higher than 0.75, including 10 with difference higher than 1). These modules are primarily in clusters 2 and 3. Notably, none of the modules shows increased abundance with microfilariae maturation. Metabolism modules for sulfur-containing amino acids are complete in *B*. *malayi* (methionine degradation and cysteine biosynthesis) and have relatively high variation in abundance (root-mean-squared Z-score across all stages ranking 3rd and 8th out of 25). In contrast, most processes associated with protein metabolism (e.g. translation, protein transport, proteasome complex) are known to not be among the most variable [72]. Interestingly, glycolysis-related modules (M00001, M00003 and M00307) were found to be relatively abundant in the late developmental stages of parasites (L3/L4 and adult male in *B*. *malayi*, L4 and adult in *A*. *suum*—*see* S1 Text). This is consistent with the L4-enrichment of glycolysis reported previously in *B*. *malayi* [75], but the free-living *C*. *elegans* showed overabundance of these modules relatively early in the life cycle (L1 to L3 and dauer entry). A higher energy requirement of parasites during L4 and adult stages may overshadow energy consumption of early development. Differentially abundant clusters of metabolic modules were also observed in *A*. *suum* and *N*. *americanus* (S13 Fig).

Previous studies based on partial transcriptomes of various nematodes have indicated differences between nematodes [37]. More specific evidence has emerged in the analyses of transcriptomes generated from comparisons of i) life-cycle stages: detection of *S*. *stercoralis* enzymes of nucleotide metabolism present in L1 but absent in the arrested L3i, is consistent with the lack of cell division and DNA replication in the L3i stage [76]; ii) different tissues: statistical enrichment of functions was reported for adult male and female *A*. *suum* when different reproductive and non-reproductive tissues were compared [77]. Similar differential expression has been confirmed in this study based on complete RNA-seq based transcriptomes.

#### Enzyme abundance and parasitism

Some nutrient requirements in parasites are supplied by the host resulting in a decreased number of required biosynthetic pathways, but a need for more active salvage pathways. For example there is no de-novo purine (A, G) biosynthetic pathway in most of the parasitic helminths studied here (with the first step—IMP biosynthesis from PRPP—being absent from most). In such cases a salvage pathway is needed to synthesize nucleotides from intermediates in the degradative pathway for nucleotides. Bases and nucleosides that are formed during degradation of RNA and DNA are also recovered and converted back to nucleotides [78]. This is an essential process for organisms and/or tissues that cannot undergo *de novo* synthesis, and such phylogenetic restrictions have been observed in our analysis.

**Fig 5:**
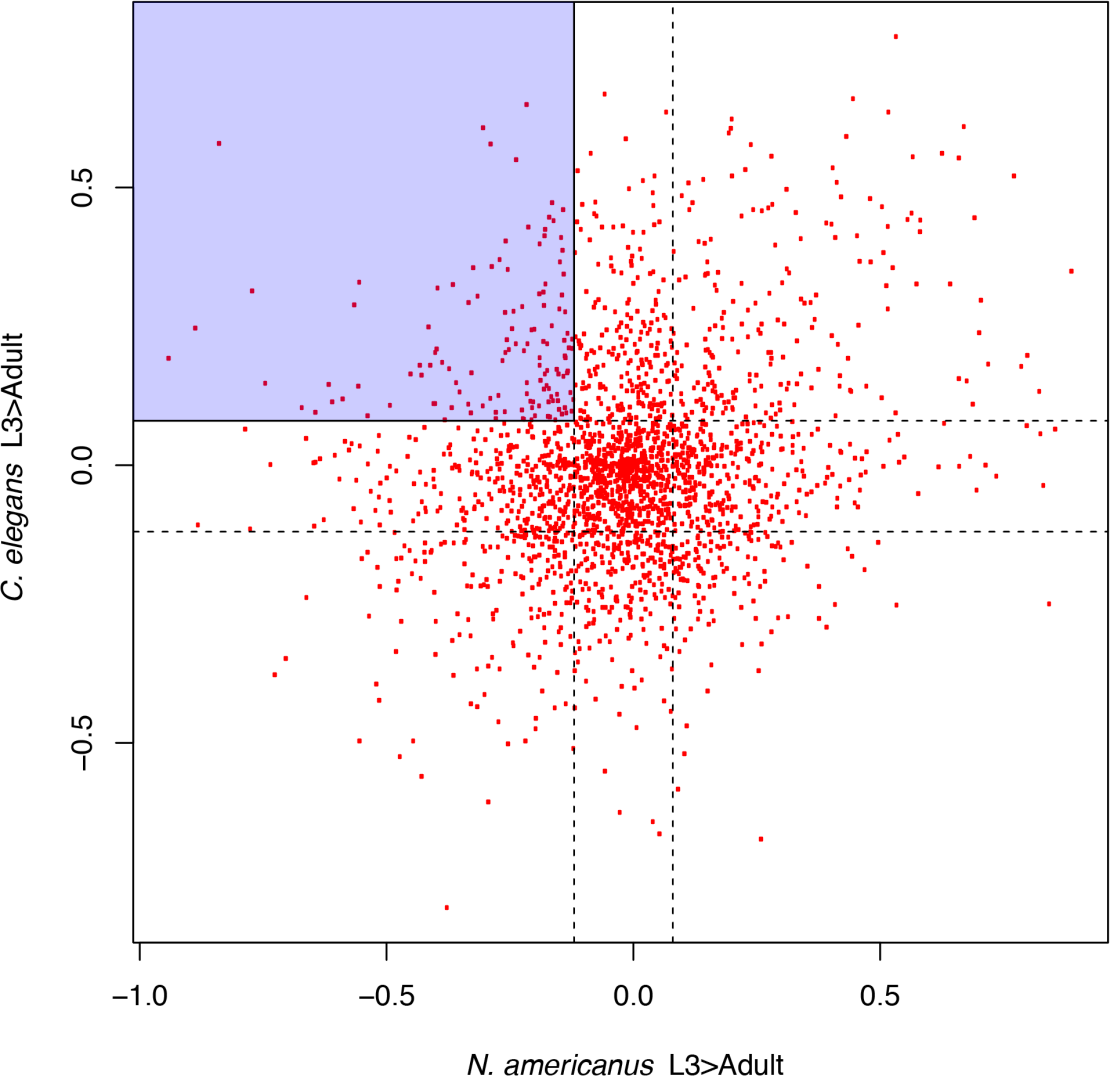
Combined analysis of differentially abundant enzymes in *Caenorhabditis elegans* and *Necator americanus*. Infective L3 stage in *N*. *americanus* is developmentally analogous to the Dauer stage of *C*. *elegans*. KOs that are overabundant in the parasitic Adult stage in the hookworm, as compared to the infective L3 stage are likely to be enriched from parasitism related enzymes, but this result is confounded by the development related KOs needed in the adult stage. We obtain a highly enriched subset of parasitism related KOs by only compiling those KOs that are underabundant in the Adult stage of *C*. *elegans*, hence, less likely to be important for the worms’ adult stage for any non-parasitism related function. The shaded region represents KOs that are among the top 25 percentile in abundance rank differential between the Dauer stage and the adult stage in *C*. *elegans* and among the bottom 25 percentile in abundance rank differential between the infective L3 stage and the adult stage in *N*. *americanus*. A total of 133 KOs lie in this region, and hence are putative parasitism-relevant KOs.

To more systematically identify putatively parasitism related metabolic enzymes, we analyzed the enzyme abundance of the human hookworm *N*. *americanus* and the relatively closely-related non-parasitic nematode *C*. *elegans*. Both species belong to the clade V of nematodes [42], with *N*. *americanus* being the closest known parasitic nematode to *C*. *elegans*. We compared the differentially abundant enzymes (KEGG KOs) encoded by the developmentally alike stages of the 2 species (*see* Methods); i.e. the dauer and adult stages of *C*. *elegans*, and the infective L3 (iL3) and adult stage of *N*. *americanus*. The iL3s in animal/human parasitic nematodes are arrested and non-feeding, similar to the dauer larvae formed by *C*. *elegans* under unfavorable conditions. This stage is of interest because of its ability to initiate infection and stimulate host immune responses [79,80]. The distribution of the KO abundances in the 2 species (Fig 5) identified a subset of KOs that is likely to be enriched for potentially parasitism related activities (Table 2). As expected, many of these categories are enriched due to homologs of genes known to have parasitism-related functions. Notable genes with possible parasitism-related functions include ABCB1 (ATP-binding cassette, subfamily B; a pump to remove toxins [81]), UGT (glucuronosyl transferase; inactivation of toxins by glucuronidation [82]), CAT (catalase; hydrogen peroxide and reactive oxygen species detoxification [83]), GAD (glutamate decarboxylase, GABA metabolism [84]), GBA (beta-glucosidase, release glucose from beta glucosides [85]) and COX15 (hydroxylation of heme O [86]). Because of the apparent enrichment of detoxification pathways among parasites, other genes in these categories are worthy of further study.

**Table 2:** Parasitism related KEGG categories.

KEGG top level category	KEGG 2nd level category	KEGG 3rd level category	hit*	background+	p-value
Organismal Systems	Digestive System	Bile secretion	4	12	0.01024
Metabolism			66	662	6.64E-06
	Glycan Biosynthesis and Metabolism	Other types of O-glycan biosynthesis	4	9	0.004594
	Xenobiotics Biodegradation and Metabolism	Drug metabolism—other enzymes	5	20	0.01115
		Metabolism of xenobiotics by cytochrome P450	4	9	0.004594
		Drug metabolism—cytochrome P450	4	10	0.006153
	Lipid Metabolism	Steroid hormone biosynthesis	4	6	0.001541
	Metabolism of Cofactors and Vitamins		14	59	3.62E-05
		Vitamin B6 metabolism	2	2	0.01744
		Porphyrin and chlorophyll metabolism	5	10	0.0009952
		Retinol metabolism	6	10	0.0001431
	Amino Acid Metabolism		24	130	2.65E-06
		Alanine, aspartate and glutamate metabolism	10	24	9.64E-06
		Cysteine and methionine metabolism	6	20	0.002571
		Glycine, serine and threonine metabolism	5	17	0.006334
	Metabolism of Other Amino Acids		9	58	0.01141
		Taurine and hypotaurine metabolism	4	5	0.0009668
		Cyanoamino acid metabolism	3	4	0.005143
		beta-Alanine metabolism	4	16	0.02289
	Energy Metabolism		16	155	0.02784
	Carbohydrate Metabolism		18	157	0.007974
		Pentose and glucuronate interconversions	6	11	0.000211
		Butanoate metabolism	4	14	0.01578
		Ascorbate and aldarate metabolism	4	15	0.01913
		Starch and sucrose metabolism	5	20	0.01115
Cellular Processes	Transport and Catabolism	Peroxisome	12	46	6.12E-05
Environmental Information Processing	Membrane Transport		4	11	0.008029
		ABC transporters	4	10	0.006153

* “hit” is the number of genes mapping to a KO of that category which is a) overexpressed in the Adult (parasitic) stage of *N*. *americanus* as compared to its iL3 stage and b) underexpressed in the Adult stage of *C*. *elegans* as compared to its Dauer stage.

+ “background” is the number of genes mapping to a KO of that category which is present in both genomes.

### Conclusions

Analyzing metabolic pathways and enzyme abundance provides important insight into the biology of an organism, especially when used in a comparative study of related species. Reconstructing entire metabolic networks of organisms has previously been done, but the sheer size and complexity of these networks means that doing a comprehensive comparison on a large set of organisms is prohibitively time and resource consuming. Furthermore, previous comparisons in parasitic worms have been based on just the enzyme content of the pathways that ignore topological information, which is too simplistic and likely to only highlight a few major differences and miss potentially useful fine details. Here, we have primarily focused on compact reaction cascades—metabolic modules—that provide some detail about an organism’s metabolic potential and illustrate how a comparison at this scale can relatively easily uncover important biological information. We provide the results for the metabolic potential of 23 organisms including 13 nematodes that include representatives from various taxonomic clades and different trophic ecologies (non-parasitic, necromenic and plant or animal parasitic). This information provides a platform for experimental follow up that could confirm *in silico* predictions we have obtained. We also indicate that certain patterns of absence of metabolic modules might be suggestive of missed genecalls, which can be used to improve genome annotations. We note i) the presence of modules that are absent from hosts but present in parasites; ii) metabolic enzyme variation between the human hookworm *N*. *americanus* and the human genome, even within metabolic modules that are complete in both the species; and iii) comparison of closely related clade V worms of genus *Caenorhabditis* and the necromenic worm *Pristionchus pacificus* showed examples of gain and loss of metabolic potentials, which is likely to provide insight into the evolution from free-living stage to parasitic dependence when the specific modular differences are explored in detail. However, we have to note that our analysis of metabolic potential is sensitive to our ability to correctly detect enzymes in proteomes of the organism under study and to the accuracy and completeness of the reference pathways we use for the reconstruction. One could advance the metabolic pathway reconstruction by improving both enzyme predictions and pathway mapping. First, one could reduce false negatives in enzyme predictions (resulting from reliance only on sequence similarity for annotation [87]) by undertaking alternate and independent methods for functional annotation, including i) utilizing the difference in sequence diversity in enzyme classes, to refine the likelihood estimation of a query protein belonging to any enzyme class [88], ii) performing Functionally Discriminating Residue recognition [89], and iii) discriminating between the functional module characteristics of different enzyme activities [90]. Using this approach on 4 nematode species, we obtained a 28% expansion of the ECs (S14 Fig). Second, in addition to KEGG, one could use some of the 133 other metabolic pathway databases available on Pathguide [91]. A majority of these are species-specific (e.g. [92]) or function-specific (e.g. [93]), but many are extensive enough to improve many organisms’ pathway reconstruction [94–96], and can produce complementary results to improve annotations [97]. Comparison of the pathways across diverse organisms could be performed to detect similar topologies [98–100]. Specifically for parasitic nematodes, the newly generated RNAseq data from 35 species and 57 stages [31,33] will provide wealth of information and pathway analysis at a level not possible before.

Finally, we report dynamic metabolic module expression between developmental stages with clusters of coexpressed modules that share overall expression profiles during development. In *B*. *malayi*, we especially note the differential metabolic dynamics of the immature and mature microfilariae. An enzyme abundance level comparison of parasitic and non-parasitic alike stages provided a set of enzymes that are likely to be implicated in parasitism-specific functions. This is the first comprehensive analysis of metabolic modules of nematodes, at a pan-Phylum level and in context of non-nematode species. The obtained results have wide applications starting from evolutionary events such as adaptations to different niches, to taxonomic restrictions that can drive pathogen control and prevention. Also, importantly, we show here that comparative study of metabolism at the level of small modules leads can lead to important biological insights without being as time and resource intensive as a study of full scale pathway reconstructions.

## Methods

### KO mapping

The proteome to KO mapping was done using a standalone implementation of KAAS [87] version 2, available from the KAAS website. We used the recommended bidirectional best hit (BBH) method for annotations with a threshold BLAST [101] mapping score of 35 bits. The eukaryotic representative set was used. *C*. *elegans* was included for annotating *C*. *elegans* also.

S1 Table shows the results for KAAS annotation of the 23 proteomes. It is also worth noting that the enzyme annotation at a primary sequence level is suboptimal for some of the species included in our analysis, for example while *Trichinella spiralis* is represented in the KEGG database, because of greater evolutionary distance with the other species in the KAAS dataset the enzyme annotation could be suboptimal.

### Modules analyzed

A module consists of 1 or more reaction steps (S1B Fig), each step consisting of 1 or more reactions that change a set of substrates into a set of products. A step is considered to be present if at least 1 set of reactions can occur in the organism i.e. there are proteins that map to at least 1 KO of each reaction that is needed to complete the reaction step.

The module definitions were downloaded from KEGG database on May 8, 2012. This has a total of 542 module definitions, 202 of which are “Pathway modules” (S1C Fig). Pathway modules are the enzymatic cascade modules that we analyzed. These modules represent 76 metabolic pathways.

We obtained a reduced set of modules based on their relevance to nematodes under study. This was done by excluding any module from the analysis set which had less than 66% of its associated KOs in all of the nematode genomes whose genome were deemed to be close to completion (all except *T*. *spiralis*, *M*. *incognita* and *P*. *pacificus*). Also, any modules with a fork leading to more than 1 branch of products (e.g. Fig 1B) were redefined as separate modules with 1 product branch per module. There were 6 such forked modules. The reduced set of modules thus obtained had 92 members.

### Ascertaining module completion

A script was written to ascertain whether a given module should be considered to be ‘complete’ within a species given the module definition, reaction data and KAAS based KO mappings of the organism’s proteome.

Some assumptions were made in order to clearly define the given data and the problem.

- The “starting substrate” and “final product” were determined simply by the order of reactions as given in the module definition. This does not work for modules that are overall cyclic, but a definition of starting and final product is not needed to ascertain their completion.
- Any compound that is not produced by any reaction in the definition of the module is considered a ‘given’—it is assumed to be present for the purpose of ascertaining module completion in all species. This includes the starting substrate but also other possible substrates (e.g. Fig 1B).

#### Cyclic modules

A cyclic module is considered complete only when every reaction stage of the cycle is present in the organism. Note that this is true only if the whole module is cyclic, rather than a module that has a cycle as a smaller constituent.

#### Non-cyclic modules

Completion of non-cyclic modules is ascertained by using a depth-first graph search method. A depth-first search [102] can potentially be time consuming, but it is effective in this case, where the largest modules are no larger than 14 reaction steps. Moreover, these steps are very often reduced by a pre-analysis step as discussed below. Since we are interested in only the presence of a path between the “final product” and the “starting substrate” of the module, the algorithm is allowed to not guarantee the discovery of the shortest possible path between these compounds. It does, however, guarantee the discovery of a path if at least 1 such path exists.

#### Network reduction by pre-analysis

In order to use simple graph-search algorithms for finding a path through a module, it is important to reduce the module to a simple directional map with just directed edges connecting nodes. For example, in the network representation of a module with substrates as nodes, the enzymes/reactions introduce conditional edges. e.g. in Fig 1C, the edge connecting 5 to 4 is contingent on the reaction C being present in the organism. Also, the edge connecting 1 to 2 is contingent not only on the presence of reaction A, but also compound 3.

As a pre-analysis step, the module network is first reduced by deleting those nodes that cannot be present based on the KO map of the proteome of the species under consideration. This reduces the complexity of the overall module network for that species and results in a simpler module with only nodes and directed edges that are no longer conditional in that organism.

#### Algorithm

The program essentially starts from the final product of the module and systematically traverses all those nodes which can produce this product by a chain of substrate-product relations. i.e. if A is a product that can be produced in a reaction (that is present in this organism’s reduced network as mentioned above) with B as 1 of the substrates, and B is similarly connected to C, then we say that A is connected to C in this network. Starting with A as the final product, we say the whole module is complete in an organism if we reach the starting substrate by this process of successive substrate discovery.

The program maintains 3 separate stacks: ‘remain’, ‘check’, and ‘connected’. These are defined as:

1. remain: nodes that remain to be checked for their connection to the final compound.
2. check: nodes that are connected to the final compound but whose immediately upstream nodes are still to be traversed.
3. connected: nodes that are connected to the final compound and whose immediately upstream nodes have been traversed.

The program logic follows the flow chart as given in Fig 1A.

### Interspecies correlation and phylogeny

The statistical package R[103] was used to calculate the Pearson correlation coefficient between species based on presence/absence of the modules that were present in at least 1 of the species under consideration. The distance metric on which the dendrogram was based, is defined as:

$$\text{d}({\text{S}}_{1},{\text{S}}_{2})=1-\text{corr}({\text{S}}_{1},{\text{S}}_{2})$$

where corr(S_1_,S_2_) is the Pearson correlation coefficient.

### Gene hunting in *N*. *americanus* assembly

The *N*. *americanus* genome assembly [43] was scanned using the genes from 11 nematode species that mapped to K00942 as query. A locally installed translated BLAST (tblastn) [104] was used and the best hit with E-value threshold of 1e-5 was reported as a match. As an alternative, we also used HMMER [105] to use a profile-HMM using the known K00942 mapped nematode genes as the query to scan the *N*. *americanus* genome assembly. Both the methods returned the same region as a match.

### Estimating module abundance

The module abundance was calculated using a “bottleneck analysis”, i.e. assuming that the overall module output will be determined by the low abundance of certain key enzyme(s), while the abundance of other higher abundance enzymes will have no impact on the output. Determining module abundance does not merely find the lowest-abundance enzymes, but also analyzes how the module topology may impact substrate concentrations throughout the module (S1D Fig).

### Module abundances and development

In order to obtain an insight into variation among stages for the modules, normalizing for the overall abundance level of the module across all stages, a Z-score was calculated based on the mean and standard deviation of the module abundances across all stages. S7 Table contains the module abundance Z-scores for all the modules potentially complete in the 4 species whose expression was analyzed. Given these Z-scores in all the available stages, the data is clustered (S8 Table) and corresponding dendrograms obtained by using heatmap.2 function of gplots R library. The default dist function is used to calculate the distance metric for clustering. In each species, a cluster threshold distance was selected such that 4 module clusters were obtained.

### Recognizing putative parasitism-related enzymes

All the KOs that were mapped to both *C*. *elegans* and *N*. *americanus* proteomes, and had an abundance value of at least 0.1 in all stages, were ranked according to their abundance in each stage (Dauer and Adult in *C*. *elegans* and the corresponding developmental stages, L3 and Adult, in *N*. *americanus*). The rank differences were calculated by subtracting Adult stage rank from the L3/Dauer stage rank. A threshold was chosen to obtain the top and bottom quartile (i.e. 25%) KOs in the lists, providing a list of KOs that are significantly higher ranked in L3/Dauer stage (the top quartile, with positive rank difference) or significantly higher ranked in Adult stage (the bottom quartile, with negative rank difference). These thresholds were 0.12 and -0.12 for *N*. *americanus*, and 0.08 and -0.1 for *C*. *elegans*.

To enrich KOs for parasitism-related functions, we examined the KOs overabundant in the parasitic Adult stage of the hookworm but underabundant in the free-living adult stage of *C*. *elegans* (i.e. overabundant in Dauer). There were 133 such KOs, which were mapped to 167 genes. KEGG categories that were significantly enriched in these KOs as compared to the whole proteome were found using Fisher’s exact test with a threshold one-tailed p-value of 0.05 (Table 2).

The KOs that were mapped to *N*. *americanus* proteome but not mapped to the *C*. *elegans* proteome were examined for KOs that are overabundant in the parasitic adult stage of *N*. *americanus* but are not encoded in the *C*. *elegans* genome. A similar analysis to the one mentioned above was done which resulted in 5 KEGG categories, only 2 of which were not part of the list obtained earlier. These are ‘Lysosome’ and ‘Glutathione Metabolism’ (italicized in Table 2).

### Data and availability

All module reconstructions generated by this study are publicly available at http://www.nematode.net/Pathway_Modules.html. The open source modDFS software for identifying complete modules can be obtained at www.nematode.net/Pathway_Modules.html and at http://sourceforge.net/projects/moddfs/.

## Supporting Information

S1 Fig Pathways, modules and module abundance. a) Module completion provides higher resolution detail than pathway completion. The 2 figures shown here are both different modules (colored in pink; left: sphingosine biosynthesis; right: sphingosine degradation) that are part of the same metabolic pathway (Sphingolipid Metabolism). As an example, if the species of interest has the sphingosine degradation module present but not sphingosine biosynthesis module, it is suggestive of sphingosine possibly being available in the environment; but the fact that the whole pathway is partially absent cannot be used for any such conclusion without looking at the details of the enzymes being absent and important metabolites that are contingent on them. The figures were made using KEGG’s online database server. b) Example of a module. This module, M00040, consists of 2 reaction steps. Reaction R01731 takes the module substrate prephenate and generates pretyrosine. Two KO ids are associated with this activity (K00832 and K15849), and hence are “alternative KOs” than can result in the completion of this reaction step. The second reaction step (pretyrosine ⇔ tyrosine) has 2 alternative reactions—R00732 and R00733, i.e. any 1 of these reactions will result in the step’s completion. Therefore, any 2 KOs, 1 from the 2 KOs of the first reaction step and 1 from the 3 KOs of the second reaction step will be sufficient for this reaction step to be complete (and hence, this two-step module to be complete). c) Module definition statistics for the KEGG database. Our work was primarily concerned with Pathway modules. d) Module abundance by bottleneck analysis. Rectangles are enzymes; ovals are compounds (substrates and products). The enzyme abundance is color coded according to the legend on bottom right. Abundance of an enzyme is taken to be the sum of all protein expression values that map to the KO corresponding to that enzyme. This is an example where the module output product (C7) can be obtained starting from the module substrate (C1) via 2 alternative pathways. Given the indicated activities, there is a separate bottleneck for both these pathways (i.e. enzyme with lowest abundance in that linear path) which together determine the abundance of C5. Note that if abundance of R6 is less than the combined abundance of the 2 pathway bottlenecks (R3 + R4), then R6 will determine the C7 output level, and hence will be the module bottleneck even though there are other lower abundance enzymes in the module (R3 and R4). This shows that module abundance estimation does not just require finding the lowest abundance enzymes in the module, but is also dependent on the module topology.
(PDF)

S2 Fig False negative annotation and completion under lenient definition. a) “Lenient” completion is defined as completion allowing for at most 1 reaction step to be absent for modules with 3 or more reaction steps. % strict completion is defined as the proportion of modules that are considered complete under the lenient definition, that are also present under strict definition. The non-worm species have a much higher % strict completion as compared to nematodes and platyhelminthes, which have strict completion values primarily in the 50–60% range. This means that for non-worm species, modules tend to either be strictly complete or they are unlikely to be missing due to just a single reaction step—consistent with their genomes and annotations being of higher quality as compared to the typical worm genome considered here. b) Missing a module due to a single KO. A rectangle represents a reaction, with rectangles for alternative reactions being on the same level (e.g. R01230 and R01231). Each of these rectangles is divided into parts of equal width based on the number of distinct KOs corresponding to the reaction (2 KOs for R00430; 1 of all others). Colored stripes inside the KO rectangles indicate the presence of the KO in the corresponding species. A linear module like M00050 needs all the steps to be available for the module to be complete. The module is absent in M. incognita because both R01230 and R01231 are unavailable to it making the second step in the cascade missing. Similarly, *N*. *americanus* and *T*. *spiralis* are missing the third step of the cascade due to lack of a KO corresponding to reaction R00332. c) A potentially missed gene in *N*. *americanus* genome assembly. 12 genes from the 11 nematode species corresponding to the missing KO (K00942) mapped with highly significant E-values to this region on contig 892 of the assembly. There is a gap in the assembly between the 2 regions that partly match the query protein sequences. Such analyses can be used for improving genome annotation.
(PDF)

S3 Fig False completion and fast evolving genes. Absent genes that are needed to complete a module by lenient definition of completion are slightly faster evolving than other genes (present genes) in the module. This analysis was done only on the nematode species in the dataset. a) The mean % identity between pairs of genes of each category is significantly different, but small. A permutation test is carried out with 1000 resamplings (with replacement) and the distribution of the difference metric (mean %id among “absent” genes—mean %id among “present” genes) is plotted. The dotted line represents actual difference (-2.8%), which is the 5^th^ percentile of the distribution. b) The “absent” set (red) contains a higher proportion of KOs that have high sequence diversity (i.e. <40% mean sequence identity among homologs) as compared to the “present” set. Using a permutation test, this subset is found to have a significant difference in mean %id of these 2 sets at a significance level of 3%.
(PDF)

S4 Fig Interspecies Pearson correlation of module completion. The highest correlation coefficients (>0.75) are almost exclusively within the *Caenorhabditis* species, flatworms, animals and plants groups. Almost no inter-group correlation is high (>0.65), with the correlation between the flatworm *C*. *sinensis* and the plant parasitic nematode *M*. *hapla* being the only exception. Conversely, very few intra-group correlation is low (<0.45), with the possibly incompletely annotated genome of *M*. *incognita* and the clade I nematode *T*. *spiralis* being the only general exceptions.
(PDF)

S5 Fig Species clustering based on module presence correlation. This is a version of Fig 2B, with complete module names replacing KEGG module IDs.
(PDF)

S6 Fig Dendrograms based on alternative analyses. a) Dendrogram based only on KO content, i.e. ignoring module topology entirely. Some significant differences as compared to the dendrogram in Fig 2B are that the hosts human and pig are now clustered with certain parasitic worms. Also, 2 plant parasites belonging to the same genus—*M*. *hapla* and *M*. *incognita—*cluster separately. b) Dendrogram based on lenient definition of module completion. Some significant differences as compared to the dendrogram based on strict definition (Fig 2B) are: 1) *C*. *sinensis* not clustering with other platyhelminthes; 2) *T*. *spiralis* clustering closer to *B*. *malayi* and *L*. *loa* as compared to other nematodes while human and pig clustering closer to nematodes. 3) *A*. *suum* and *N*. *americanus* not clustering with *Caenorhabditis* species. This suggests that the lenient definition might not be a reliable indicator of module completion, with the lower number of false positives obtained at the cost of possibly lower positive predictive value. Hence, we recommend using strict definition of completion for ascertaining module completion and lenient definition for ascertaining absence of a module with high confidence.
(PDF)

S7 Fig Module exclusion or conservation based on phylogeny and parasitism. a) Venn showing non-exclusion from a species group i.e. “present in at least 1 species of the group”. A total of 77 modules are present in at least 1 species. b) Conservation throughout a group i.e. “present in all the species of the group”. 6 modules are present in all the species. c) Non-exclusion among nematode groups. A total of 51 modules are present in at least 1 nematode. d) Conservation throughout a nematode group. 8 modules are present in all the nematodes.
(PDF)

S8 Fig Metabolic potential of *P*. *pacificus* and *Caenorhabditis* species. a) Red indicates absence of the module and green indicate presence (under the strict completion definition). b) Comparison of metabolic potential of *P*. *pacificus* and *Caenorhabditis* species. The core metabolic modules for the genus *Caenorhabditis* consists of 29 metabolic modules. Relatively fewer modules are complete in a smaller subset of the 4 species, with *C*. *remanei* observed to be notably distinct from others. c) Comparison of module potential distinction between the genus *Caenorhabditis* and *P*. *pacificus*. Red circle represents the set of modules that are present in all 4 *Caenorhabditis* species (a total of 29, as mentioned in (b) above). The green circle represents modules present in *P*. *pacificus* proteome. Out of the 6 modules absent in *P*. *pacificus* but present in the *Caenorhabditis* core modules, 2 are absent in *P*. *pacificus* even under lenient definition. Similarly, 2 modules are absent in all *Caenorhabditis* worms even under lenient definition, but present in *P*. *pacificus* under strict definition.
(PDF)

S9 Fig KEGG modules miss some interesting details. It is known that certain worms that are included in our study show extensive fucosylation [106]. However, this insight is missed by our analysis because the relevant part (shown within red box) of the pathway are not covered by any module definition. The pink boxes represent the only part of the pathway that is part of a module definition (M00114), and can be analyzed by the current implementation of our method.
(PDF)

S10 Fig N- and O-glycans of helminths and humans. a. N-glycan variants among helminths are represented as single elaborated structures—in living organisms, N-glycan structures are heterogeneous. Glycosyltransferases create bonds between monosaccharides, given as configuration (alpha α or beta β) along with linkage position (2,3,4,6). Our metabolic module analysis is consistent with the backbone structure of N-glycans (chitobiose trimannose). Consistent with previous work, nematoda and platyhelminths under study lack sialyltransferases found in mammals. Previous biochemical analysis of helminth N-glycans has demonstrated phosphorylcholine and xylose modifications not found in humans (boxed). Immunologically-important glycosylation patterns for parasitic worms are: Lewis X (Galβ1–4[Fucα1–3]GlcNAc-) and LDNF (GalNAcβ1–4[Fucα1–3]GlcNAc-), as described in the text. *b*. *O-glycans of helminths*. Mammalian O-glycans are frequently sialylated (α2,3 or α2,6). Elaborated mammalian glycans built on Core 2 and Core 4 are depicted here, but other sialylated structures are also synthesized. Carbohydrates lacking sialic acid, including T antigen and Tn antigen (right) have been detected experimentally in multiple helminths. In contrast to human, O-glycans of nematodes and flatworms can be glucuronidated and methylated—these features were not explicitly detected by computational analysis using KEGG modules.
(PDF)

S11 Fig Comparison of KOs encoded in *N*. *americanus* and *H*. *sapiens* genomes. While host or parasite specific KOs form an obvious basis of alternative metabolism, even the shared KOs have a significant fraction that are associated with proteins with indels that can be used to specifically target the parasite.
(PDF)

S12 Fig Heatmaps showing clustering of modules and developmental stages based on module abundance patterns in *C*. *elegans* and *B*.*malayi*. The red line divides modules into 4 clusters that share developmental stage abundance profiles. The heatmap rows of these clusters are separated by a black bar for clarity. a) *C*. *elegans*. Interestingly, while young adult stage clusters with L2, L3 and L4 stages, Adult stage seems pretty close to the Dauer stages. This is primarily due to an overall depression in module abundances in Adult stage which is similar to the overall trend for Dauer stages. b) *B*. *malayi*. Interestingly, Adult male and female stages cluster with immature and mature microfilariae respectively. Also, L3 is especially notable for almost uniform overabundance, which is in sharp contrast to mature microfilariae which has almost uniform underabundance.
(PDF)

S13 Fig Module abundance dynamics in *A*. *suum* and *N*. *americanus*. a) A Heatmap showing clustering of modules and developmental stages based on module abundance patterns in *A*. *suum*. The adult stage abundances have been combined here to aid in obtaining clusters more relevant to developmental differences. There are clear differences in stages here, with some modules very clearly showing high overabundance in only 1 of the developmental stages (especially L3_lung and Adult). The red line divides modules into 4 clusters that share abundance profiles in these developmental stages. The heatmap rows of these clusters are separated by a black bar for clarity. b) Developmental stage based metabolic profiles *A*. *suum*. The cluster characteristics are discussed in S1 Text. These plots are analogous to those presented in Fig 4 for *C*. *elegans* and *B*. *malayi*. c) Module abundance ratios (in log2 units) for *Necator americanus* metabolic modules. Modules 56a and 57 have 0 Adult/L3 abundance ratio, and there bars on the graph are indicative of-inf. 17 out of 36 modules have a significant difference between L3 and adult abundances (|ln(Adult/L3)|>1), with 10 modules being overabundant in Adult stage and 7 in the infective L3 stage.
(PDF)

S14 Fig Improving enzyme annotation by using alternative annotation methods. a) Using the 3 methods expands the annotated enzyme set by an average of 33.1% w.r.t. annotations based only on KAAS in *C*. *elegans*. Allowing only those additional annotations that are supported by at least 2 methods expands our annotation set for *C*. *elegans* by 4.9%. (b-d) Including 3 parasitic nematodes (b: *T*. *spiralis*, c: *N*. *americanus*, d: *P*. *pacificus)* resulted in the mean expansion of their enzyme activity detection by 27.8%. Allowing only new high confidence annotation (as determined by their confirmation by at least 2 of the methods) expands our enzyme annotation by 3.7%.
(PDF)

S1 Table Deduced proteomes of species studied. (XLSX)

S2 Table Module complete under strict and lenient definition. (XLSX)

S3 Table Blast results for *Pristionchus pacificus* gene GENEPREDICTION_SNAP300000079726: a potential case for horizontal gene transfer. (XLSX)

S4 Table Comparison of parasite and host reactions. These include all cases where either a parasite-exclusive enzyme or a host-exclusive enzyme is found in a module that is complete in both the parasite and the host. Reactions with mutually exclusive KOs without any shared KOs are highlighted in red. Cases with alternative reactions achieving the completion of the same reaction step are also reported at the end of each host-parasite table and highlighted in bold text. The comparison code has "S" for at least 1 shared KO (or reaction in case of alternative reaction reporting), "H" for at least 1 KO/reaction that is host specific and "P" for at least 1 KO/reaction that is parasite specific.
(XLSX)

S5 Table Insertions and deletions specific to *N*. *americanus* genes compared to human orthologs. The genes mapping to KOs common to both proteomes have those KOs shown in column C.
(XLSX)

S6 Table Species whose expression is analyzed under different developmental stages. (XLSX)

S7 Table Abundance Z-scores for modules in four nematodes. (XLSX)

S8 Table Development stage based abundance profile clusters of metabolic modules. (XLSX)

S1 Text Metabolic dynamics in parasitic nematodes. (DOCX)
