# Supplementary figures and images for: Pan-phylum Comparison of Nematode Metabolic Potential
Source: PLoS Negl Trop Dis. 2015 May 22;9(5):e0003788. doi: 10.1371/journal.pntd.0003788 (PMC4441503; doi:10.1371/journal.pntd.0003788)

a)

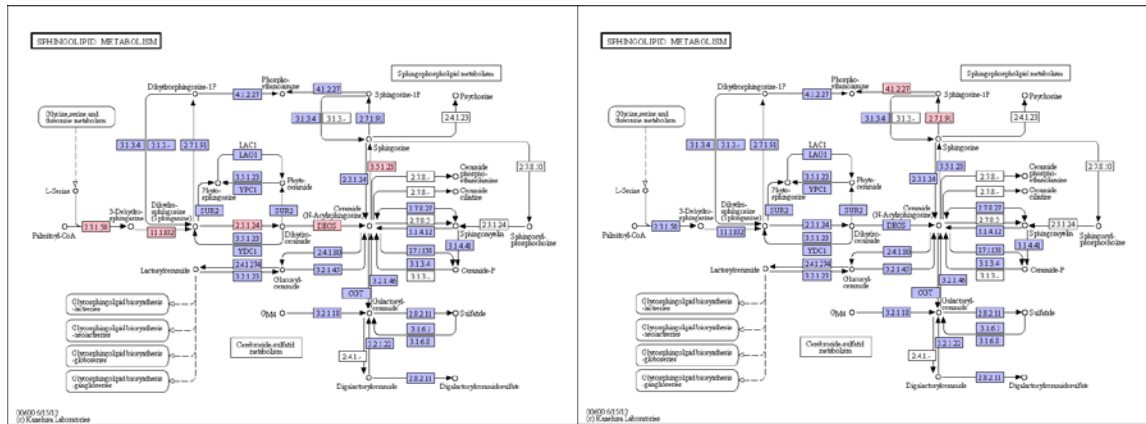

b)

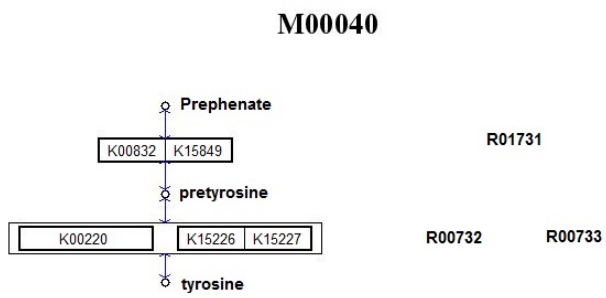

c)

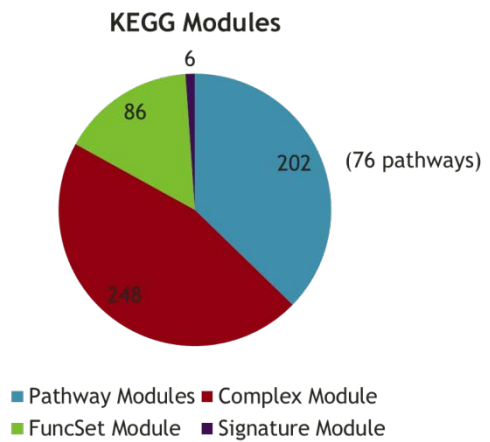

d)

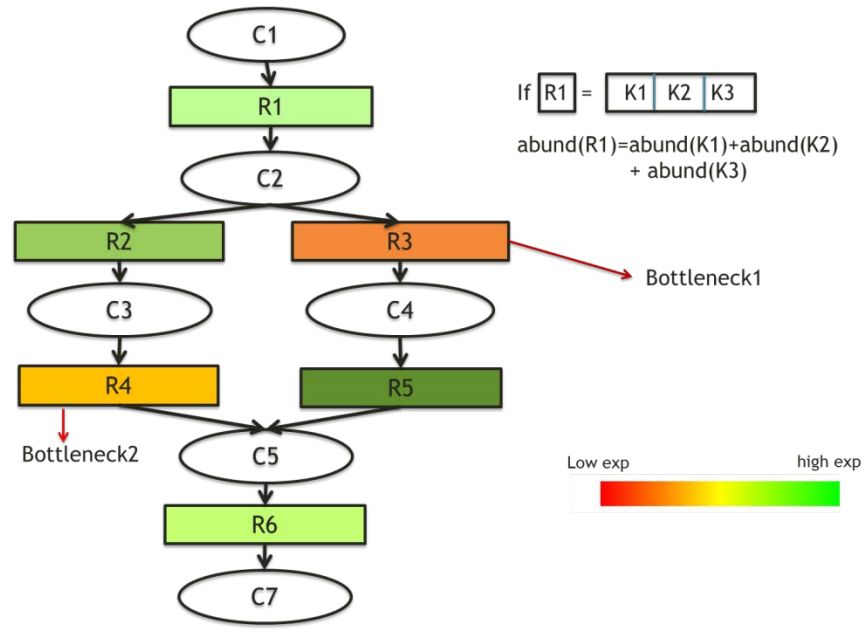

Supplement: S1 Fig — a) Module completion provides higher resolution detail than pathway completion. The 2 figures shown here are both different modules (colored in pink; left: sphingosine biosynthesis; right: sphingosine degradation) that are part of the same metabolic pathway (Sphingolipid Metabolism). As an example, if the species of interest has the sphingosine degradation module present but not sphingosine biosynthesis module, it is suggestive of sphingosine possibly being available in the environment; but the fact that the whole pathway is partially absent cannot be used for any such conclusion without looking at the details of the enzymes being absent and important metabolites that are contingent on them. The figures were made using KEGG’s online database server. b) Example of a module. This module, M00040, consists of 2 reaction steps. Reaction R01731 takes the module substrate prephenate and generates pretyrosine. Two KO ids are associated with this activity (K00832 and K15849), and hence are “alternative KOs” than can result in the completion of this reaction step. The second reaction step (pretyrosine ⇔ tyrosine) has 2 alternative reactions—R00732 and R00733, i.e. any 1 of these reactions will result in the step’s completion. Therefore, any 2 KOs, 1 from the 2 KOs of the first reaction step and 1 from the 3 KOs of the second reaction step will be sufficient for this reaction step to be complete (and hence, this two-step module to be complete). c) Module definition statistics for the KEGG database. Our work was primarily concerned with Pathway modules. d) Module abundance by bottleneck analysis. Rectangles are enzymes; ovals are compounds (substrates and products). The enzyme abundance is color coded according to the legend on bottom right. Abundance of an enzyme is taken to be the sum of all protein expression values that map to the KO corresponding to that enzyme. This is an example where the module output product (C7) can be obtained starting from the module substrate (C1) [file pntd.0003788.s001.pdf]

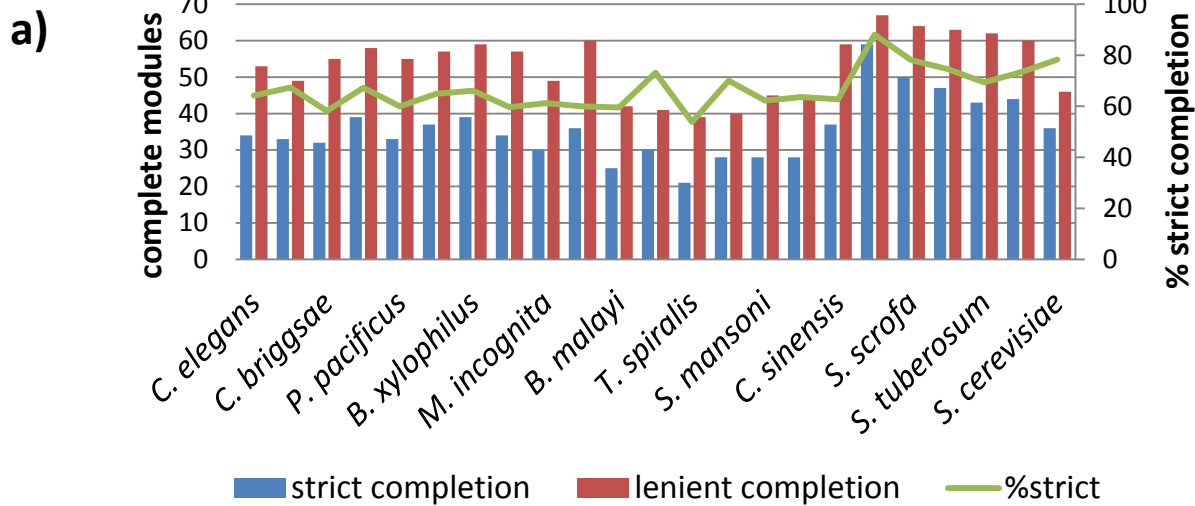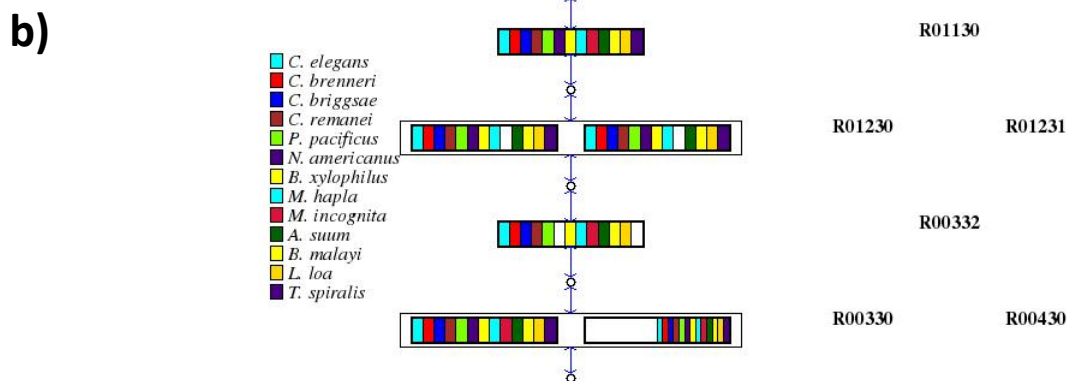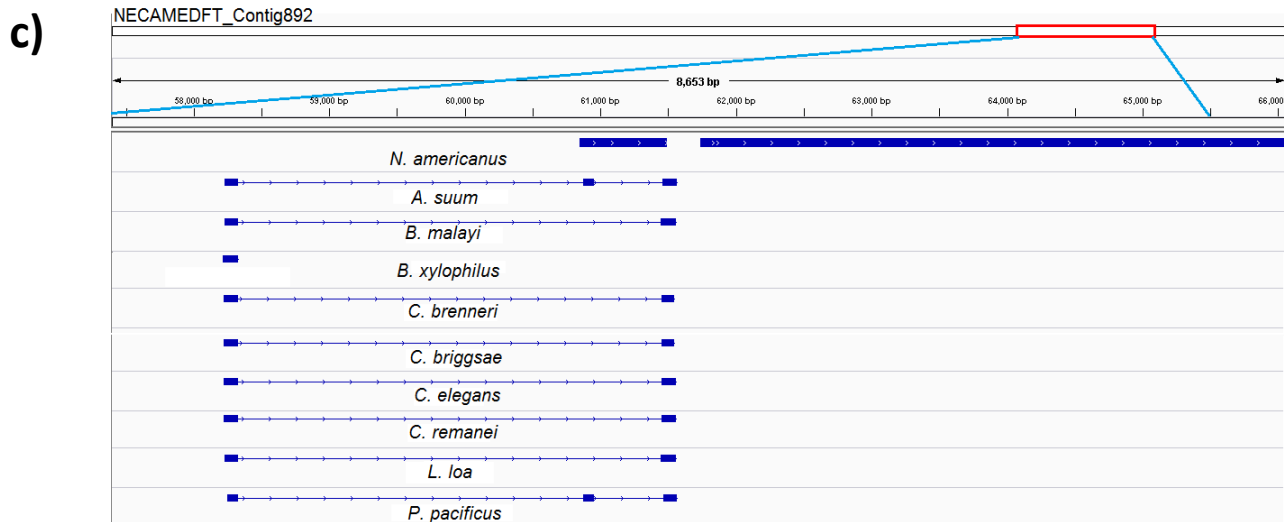

Supplement: S2 Fig — a) “Lenient” completion is defined as completion allowing for at most 1 reaction step to be absent for modules with 3 or more reaction steps. % strict completion is defined as the proportion of modules that are considered complete under the lenient definition, that are also present under strict definition. The non-worm species have a much higher % strict completion as compared to nematodes and platyhelminthes, which have strict completion values primarily in the 50–60% range. This means that for non-worm species, modules tend to either be strictly complete or they are unlikely to be missing due to just a single reaction step—consistent with their genomes and annotations being of higher quality as compared to the typical worm genome considered here. b) Missing a module due to a single KO. A rectangle represents a reaction, with rectangles for alternative reactions being on the same level (e.g. R01230 and R01231). Each of these rectangles is divided into parts of equal width based on the number of distinct KOs corresponding to the reaction (2 KOs for R00430; 1 of all others). Colored stripes inside the KO rectangles indicate the presence of the KO in the corresponding species. A linear module like M00050 needs all the steps to be available for the module to be complete. The module is absent in M. incognita because both R01230 and R01231 are unavailable to it making the second step in the cascade missing. Similarly, N. americanus and T. spiralis are missing the third step of the cascade due to lack of a KO corresponding to reaction R00332. c) A potentially missed gene in N. americanus genome assembly. 12 genes from the 11 nematode species corresponding to the missing KO (K00942) mapped with highly significant E-values to this region on contig 892 of the assembly. There is a gap in the assembly between the 2 regions that partly match the query protein sequences. Such analyses can be used for improving genome annotation. (PDF) [file pntd.0003788.s002.pdf]

**a)**

**distribution of difference of means between permuted sets**

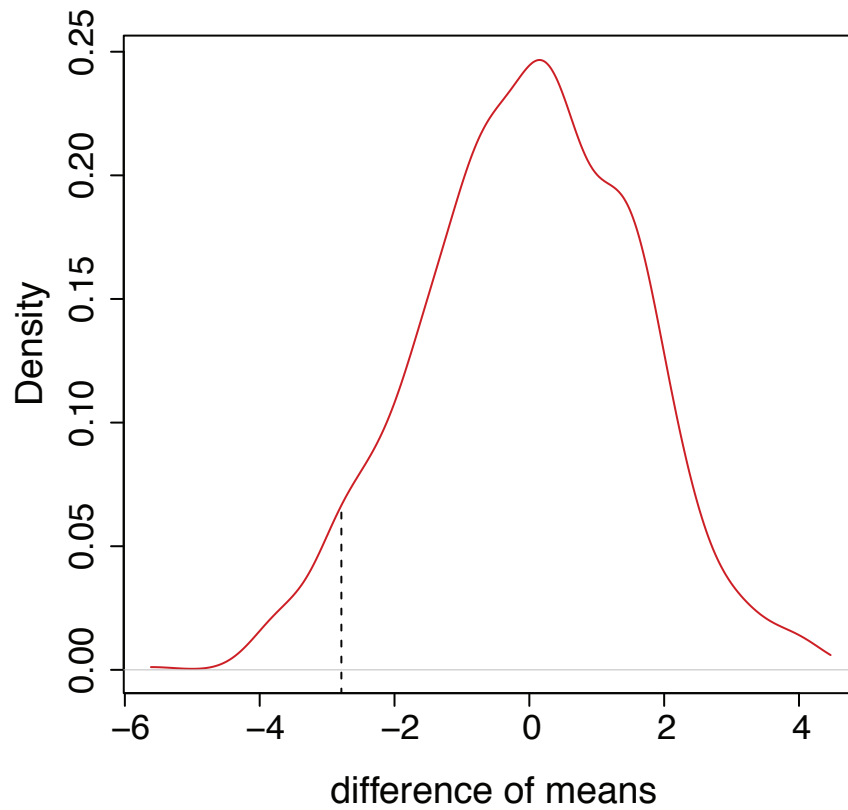

**b)**

**%id of "absent" vs "present" KOs**

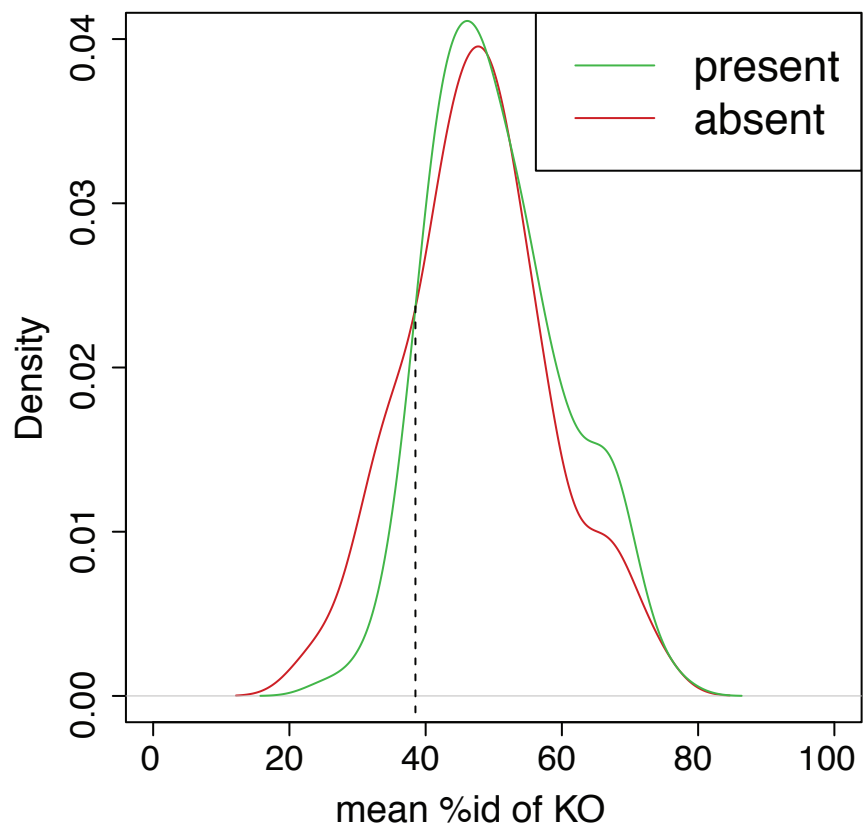

Supplement: S3 Fig — Absent genes that are needed to complete a module by lenient definition of completion are slightly faster evolving than other genes (present genes) in the module. This analysis was done only on the nematode species in the dataset. a) The mean % identity between pairs of genes of each category is significantly different, but small. A permutation test is carried out with 1000 resamplings (with replacement) and the distribution of the difference metric (mean %id among “absent” genes—mean %id among “present” genes) is plotted. The dotted line represents actual difference (-2.8%), which is the 5th percentile of the distribution. b) The “absent” set (red) contains a higher proportion of KOs that have high sequence diversity (i.e. <40% mean sequence identity among homologs) as compared to the “present” set. Using a permutation test, this subset is found to have a significant difference in mean %id of these 2 sets at a significance level of 3%. (PDF) [file pntd.0003788.s003.pdf]

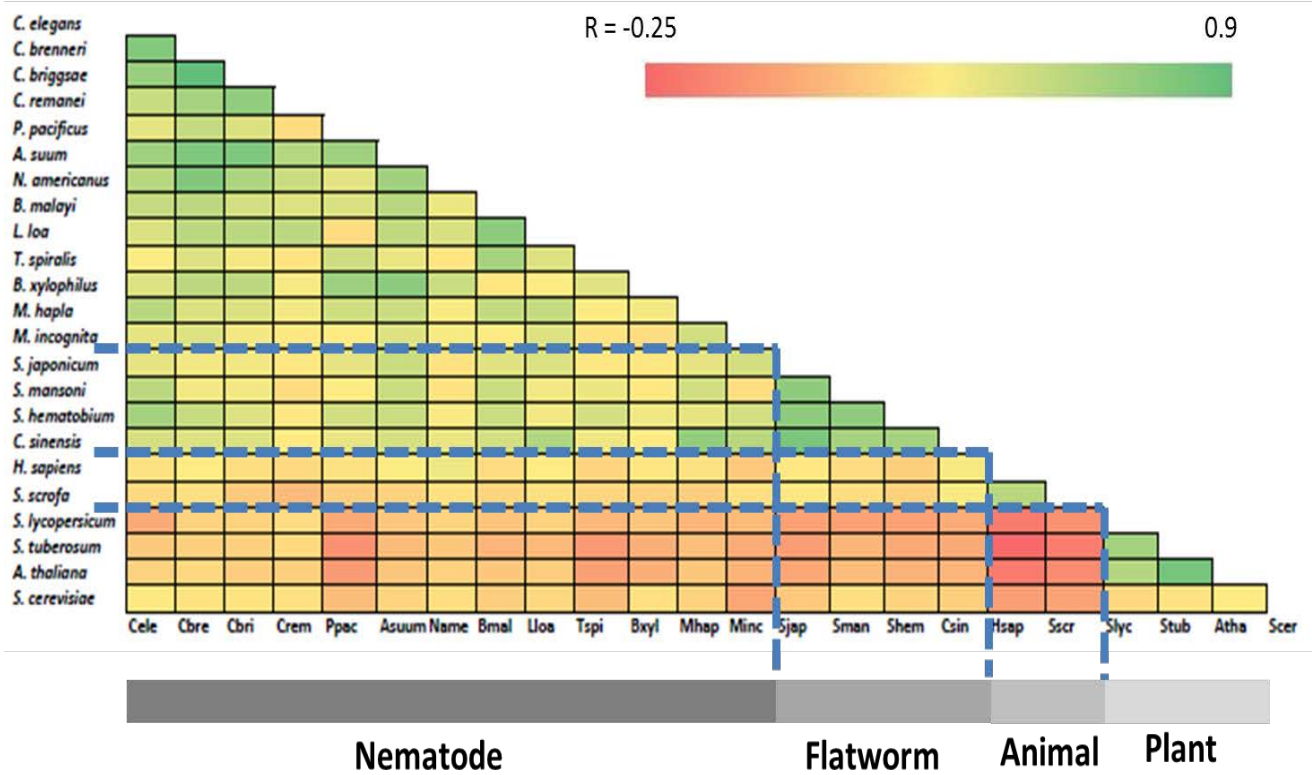

Supplement: S4 Fig — The highest correlation coefficients (>0.75) are almost exclusively within the Caenorhabditis species, flatworms, animals and plants groups. Almost no inter-group correlation is high (>0.65), with the correlation between the flatworm C. sinensis and the plant parasitic nematode M. hapla being the only exception. Conversely, very few intra-group correlation is low (<0.45), with the possibly incompletely annotated genome of M. incognita and the clade I nematode T. spiralis being the only general exceptions. (PDF) [file pntd.0003788.s004.pdf]

**a)**

— 0.1

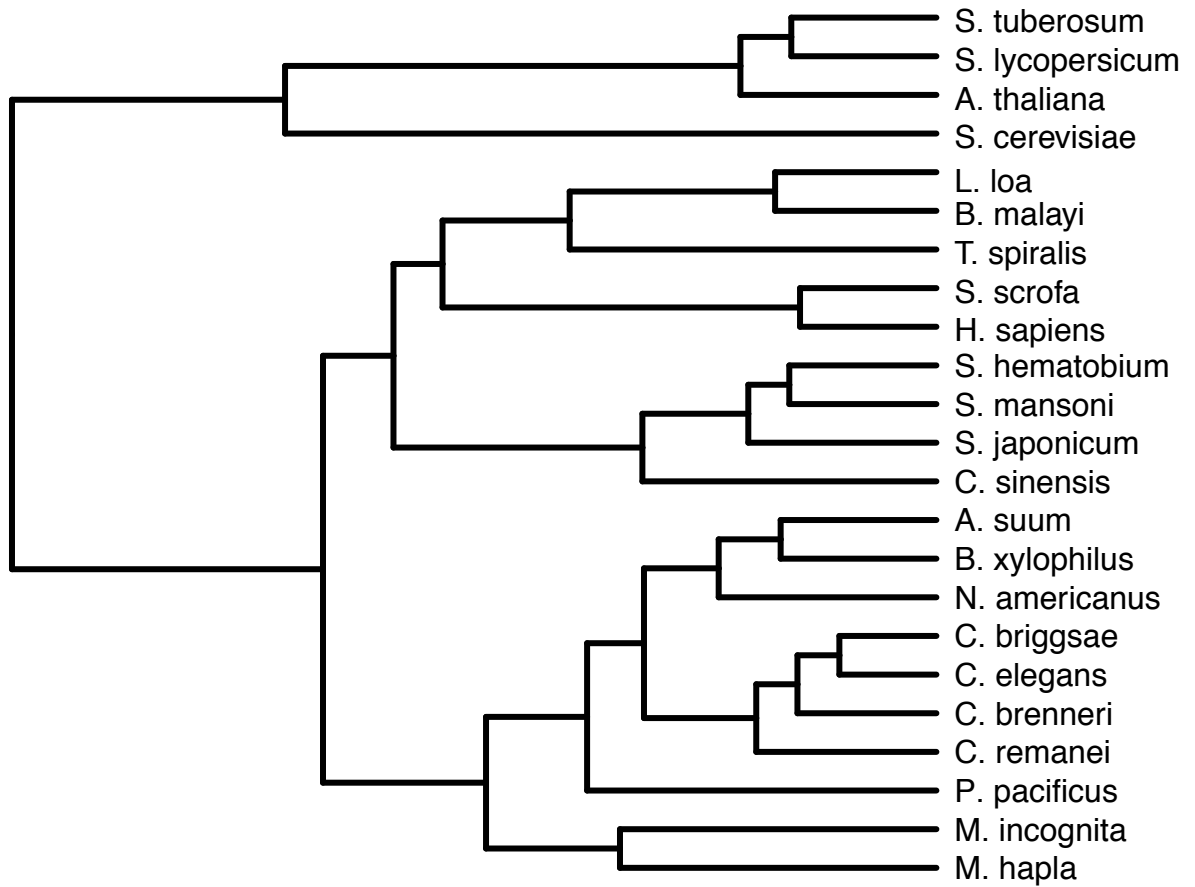

**b)**

— 0.1

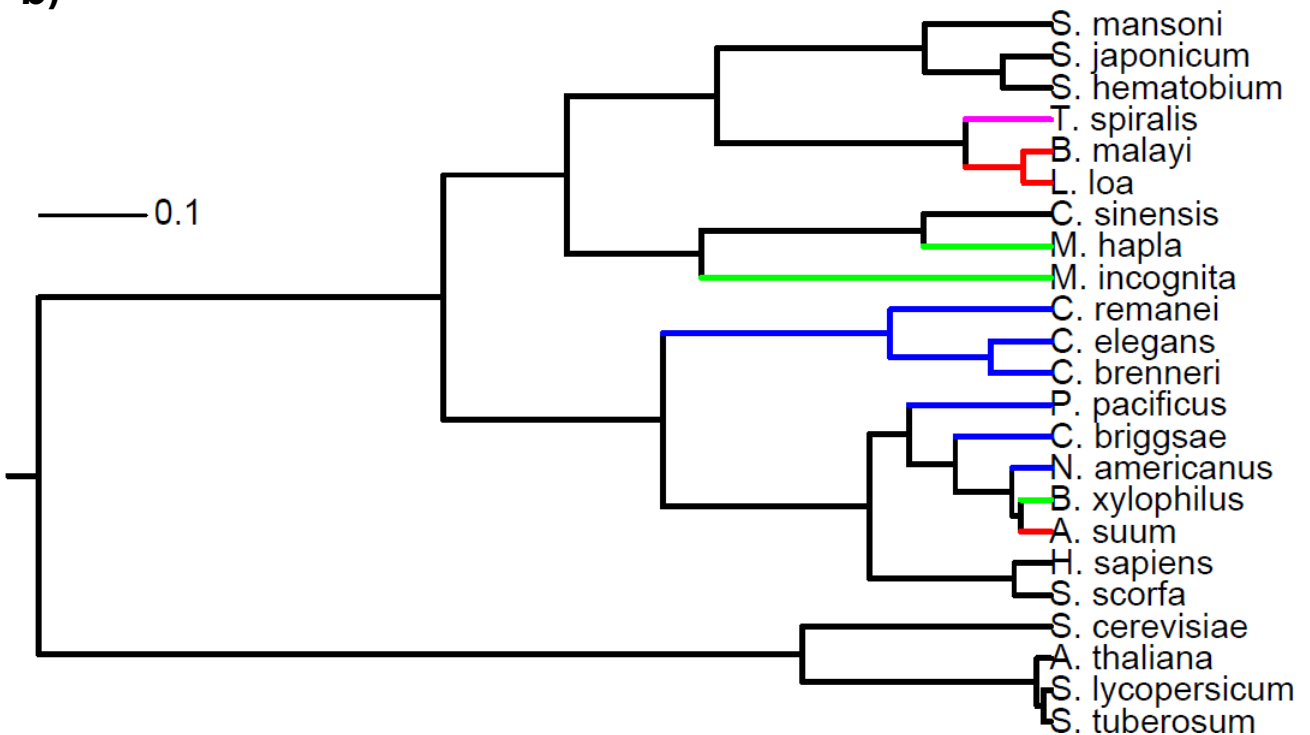

Supplement: S6 Fig — a) Dendrogram based only on KO content, i.e. ignoring module topology entirely. Some significant differences as compared to the dendrogram in Fig 2B are that the hosts human and pig are now clustered with certain parasitic worms. Also, 2 plant parasites belonging to the same genus—M. hapla and M. incognita—cluster separately. b) Dendrogram based on lenient definition of module completion. Some significant differences as compared to the dendrogram based on strict definition (Fig 2B) are: 1) C. sinensis not clustering with other platyhelminthes; 2) T. spiralis clustering closer to B. malayi and L. loa as compared to other nematodes while human and pig clustering closer to nematodes. 3) A. suum and N. americanus not clustering with Caenorhabditis species. This suggests that the lenient definition might not be a reliable indicator of module completion, with the lower number of false positives obtained at the cost of possibly lower positive predictive value. Hence, we recommend using strict definition of completion for ascertaining module completion and lenient definition for ascertaining absence of a module with high confidence. (PDF) [file pntd.0003788.s006.pdf]

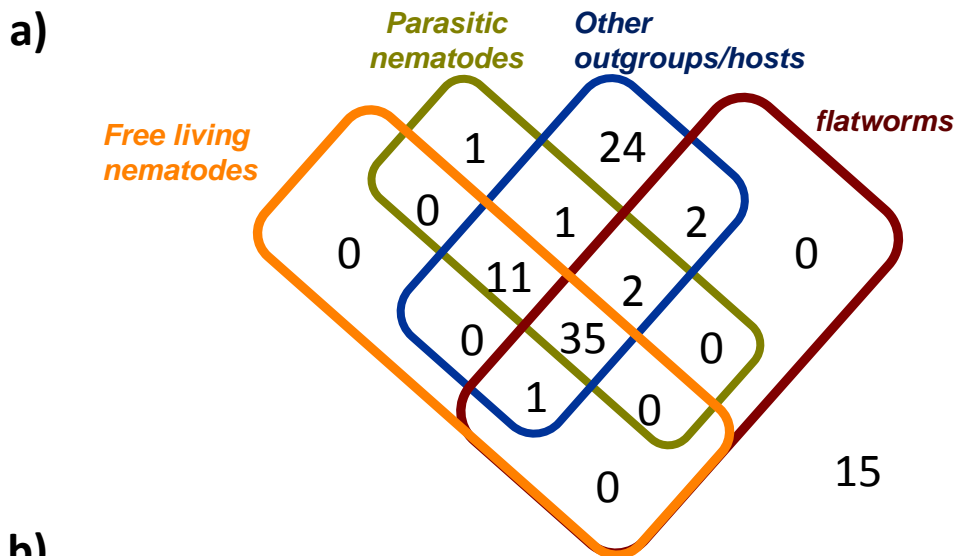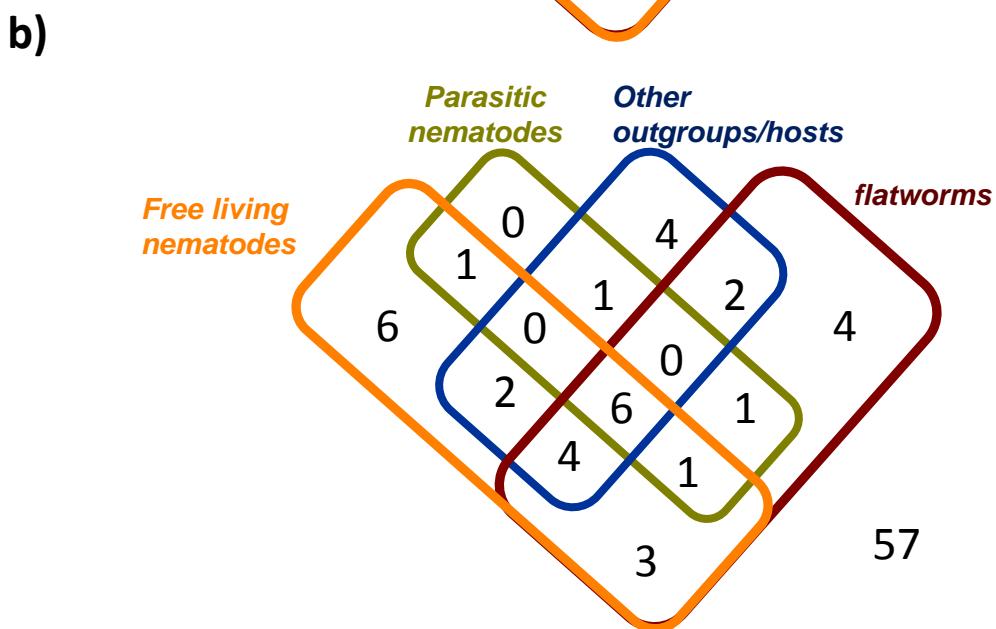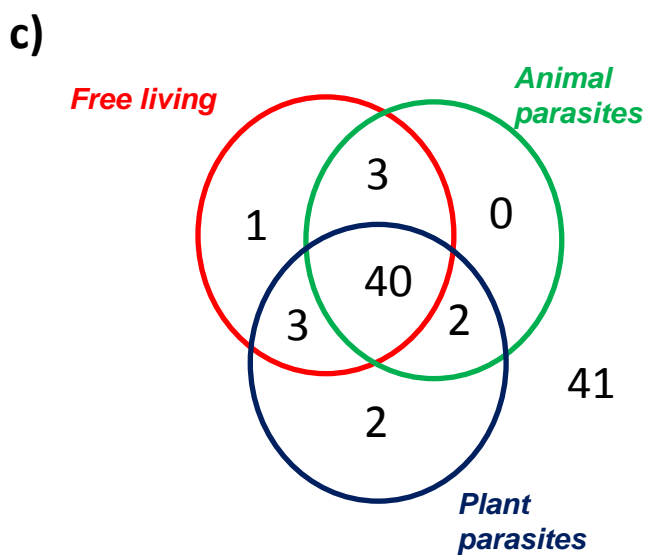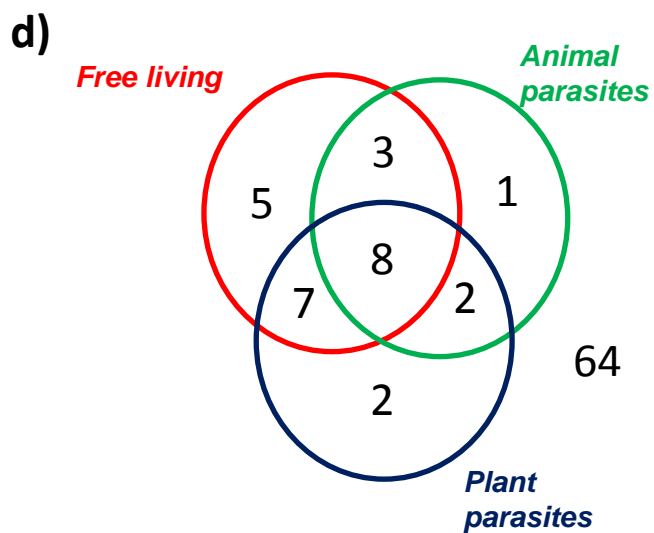

Supplement: S7 Fig — a) Venn showing non-exclusion from a species group i.e. “present in at least 1 species of the group”. A total of 77 modules are present in at least 1 species. b) Conservation throughout a group i.e. “present in all the species of the group”. 6 modules are present in all the species. c) Non-exclusion among nematode groups. A total of 51 modules are present in at least 1 nematode. d) Conservation throughout a nematode group. 8 modules are present in all the nematodes. (PDF) [file pntd.0003788.s007.pdf]

a)

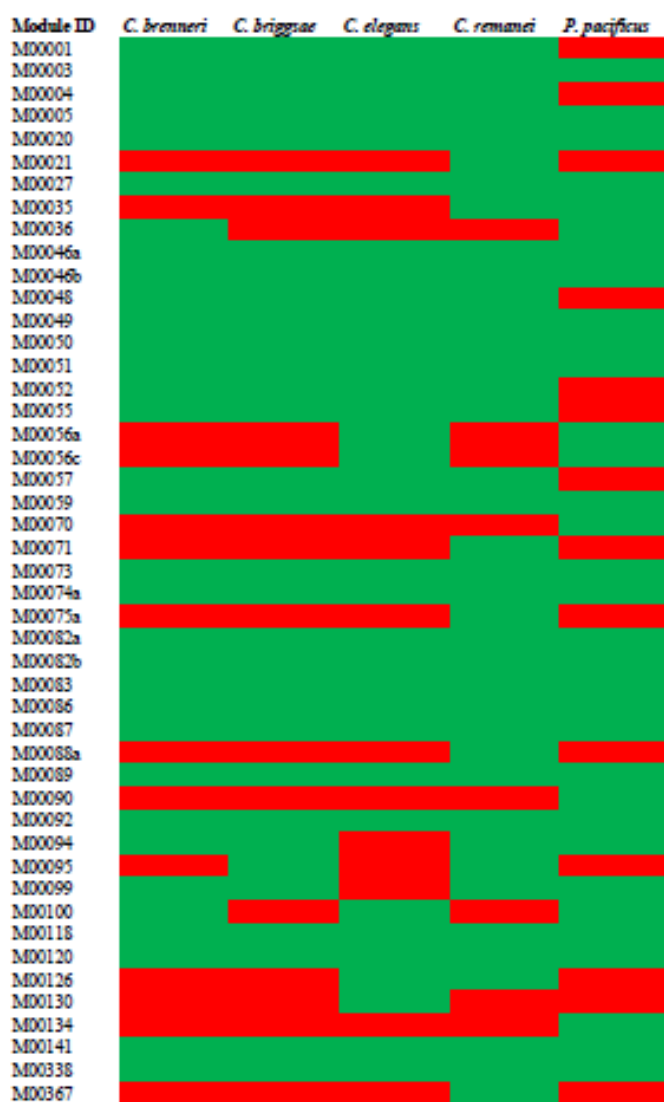

b)

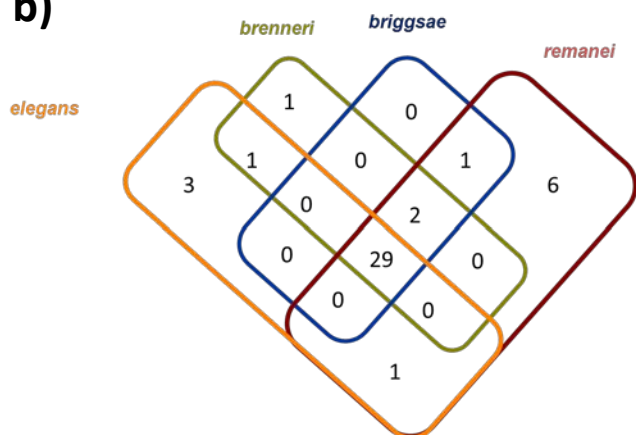

c)

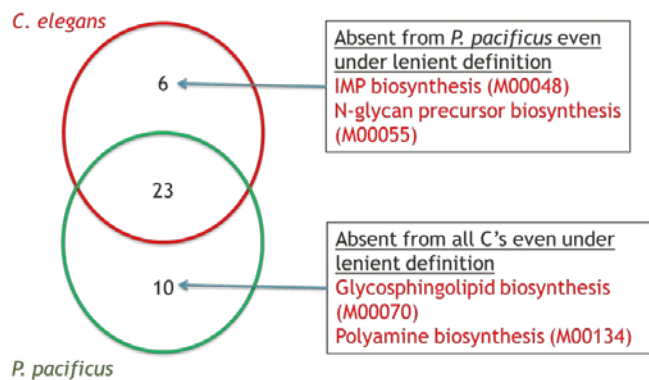

Supplement: S8 Fig — a) Red indicates absence of the module and green indicate presence (under the strict completion definition). b) Comparison of metabolic potential of P. pacificus and Caenorhabditis species. The core metabolic modules for the genus Caenorhabditis consists of 29 metabolic modules. Relatively fewer modules are complete in a smaller subset of the 4 species, with C. remanei observed to be notably distinct from others. c) Comparison of module potential distinction between the genus Caenorhabditis and P. pacificus. Red circle represents the set of modules that are present in all 4 Caenorhabditis species (a total of 29, as mentioned in (b) above). The green circle represents modules present in P. pacificus proteome. Out of the 6 modules absent in P. pacificus but present in the Caenorhabditis core modules, 2 are absent in P. pacificus even under lenient definition. Similarly, 2 modules are absent in all Caenorhabditis worms even under lenient definition, but present in P. pacificus under strict definition. (PDF) [file pntd.0003788.s008.pdf]

## FRUCTOSE AND MANNOSE METABOLISM

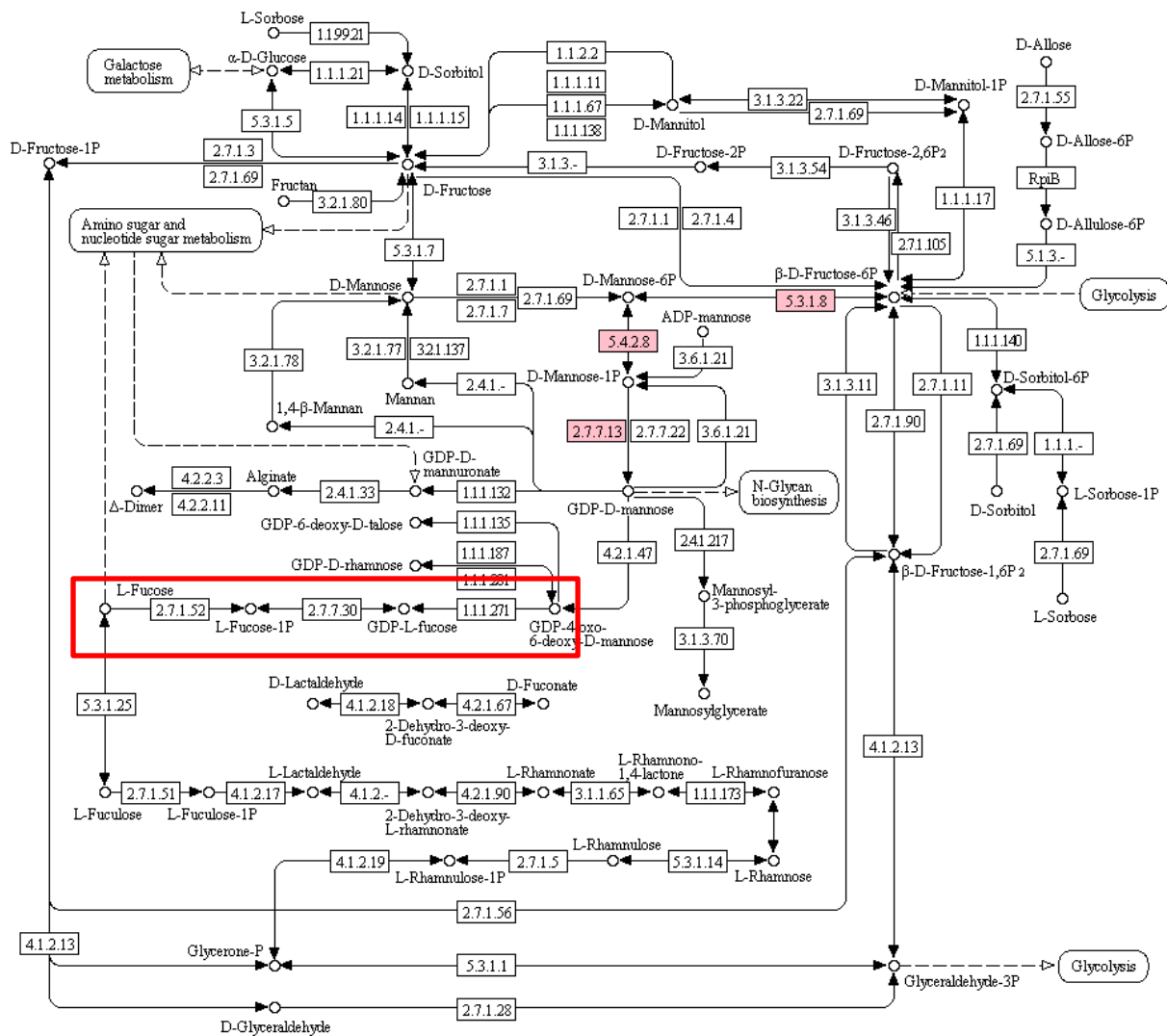

Supplement: S9 Fig — It is known that certain worms that are included in our study show extensive fucosylation [106]. However, this insight is missed by our analysis because the relevant part (shown within red box) of the pathway are not covered by any module definition. The pink boxes represent the only part of the pathway that is part of a module definition (M00114), and can be analyzed by the current implementation of our method. (PDF) [file pntd.0003788.s009.pdf]

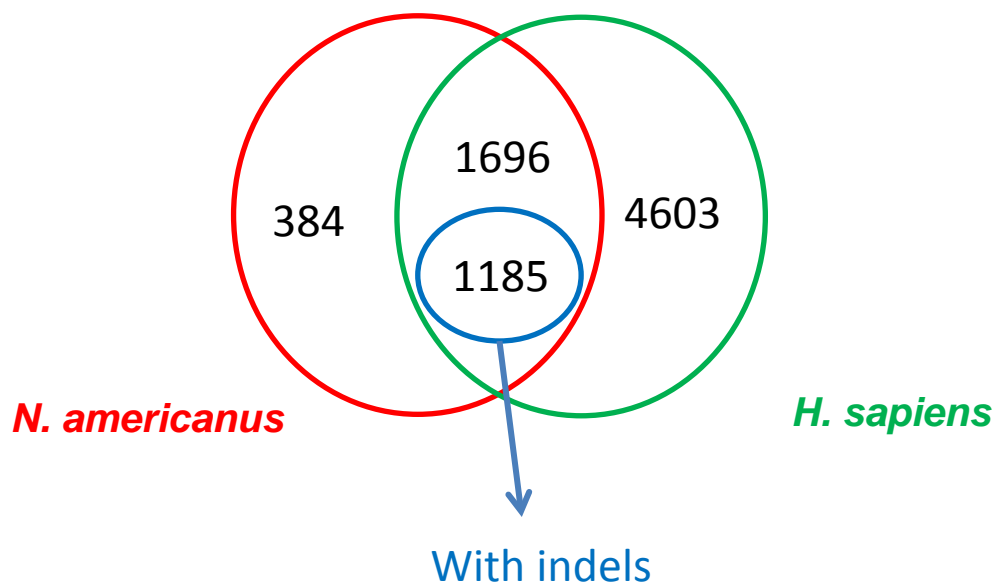

Supplement: S11 Fig — While host or parasite specific KOs form an obvious basis of alternative metabolism, even the shared KOs have a significant fraction that are associated with proteins with indels that can be used to specifically target the parasite. (PDF) [file pntd.0003788.s011.pdf]

a) C. elegans

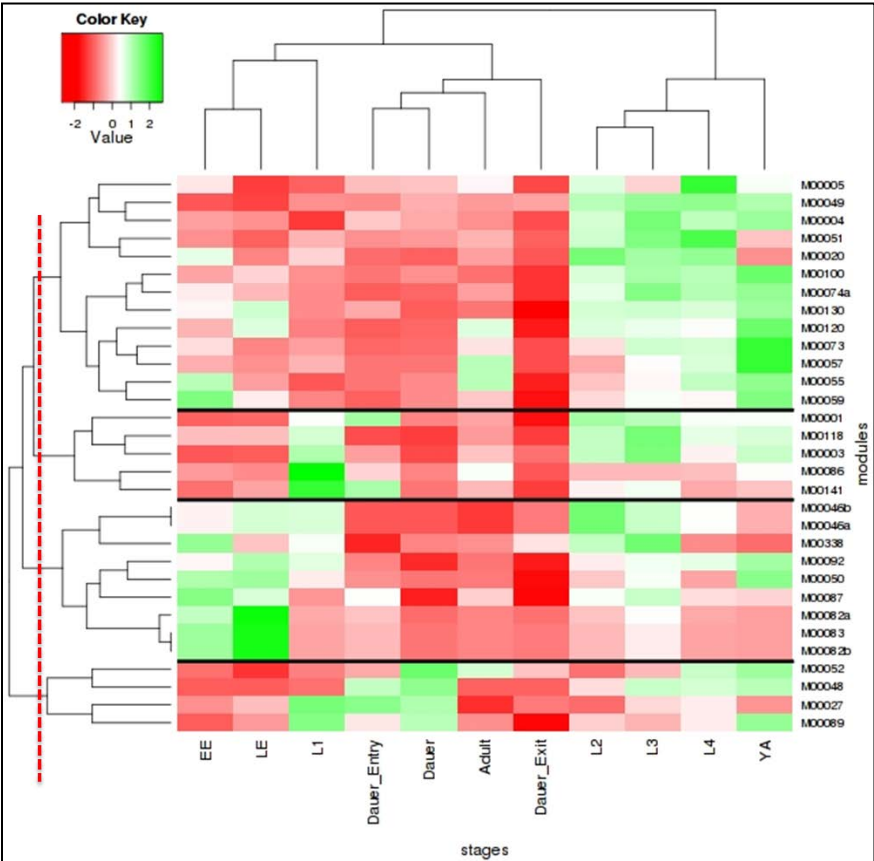

b) B. malayi

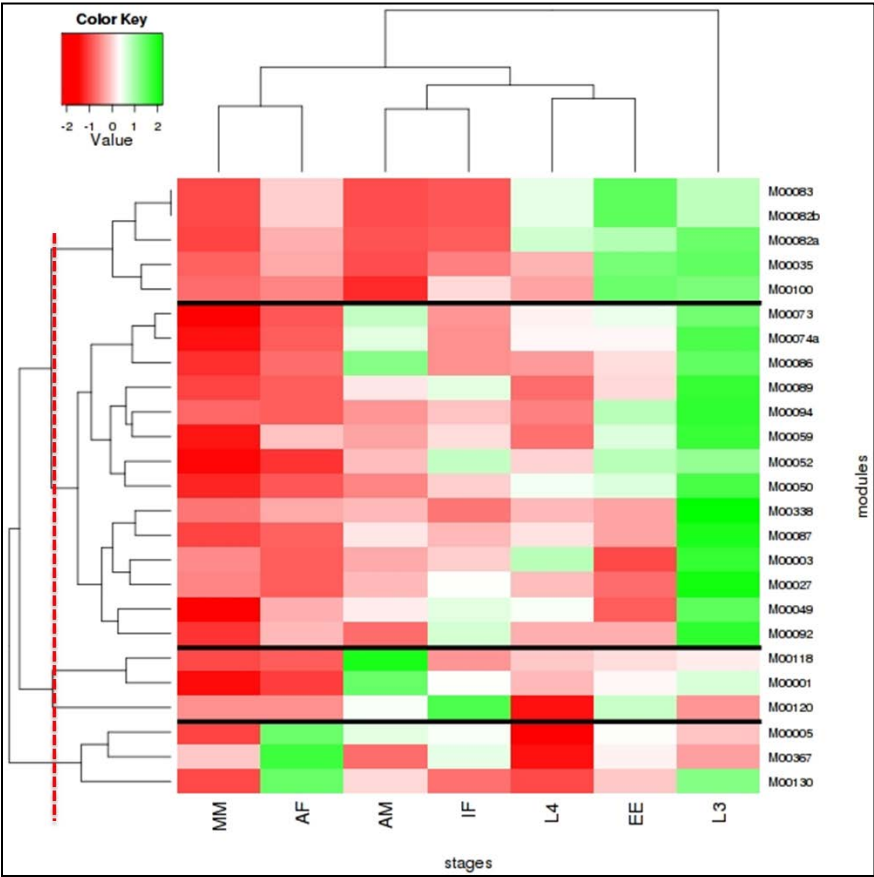

Supplement: S12 Fig — The red line divides modules into 4 clusters that share developmental stage abundance profiles. The heatmap rows of these clusters are separated by a black bar for clarity. a) C. elegans. Interestingly, while young adult stage clusters with L2, L3 and L4 stages, Adult stage seems pretty close to the Dauer stages. This is primarily due to an overall depression in module abundances in Adult stage which is similar to the overall trend for Dauer stages. b) B. malayi. Interestingly, Adult male and female stages cluster with immature and mature microfilariae respectively. Also, L3 is especially notable for almost uniform overabundance, which is in sharp contrast to mature microfilariae which has almost uniform underabundance. (PDF) [file pntd.0003788.s012.pdf]

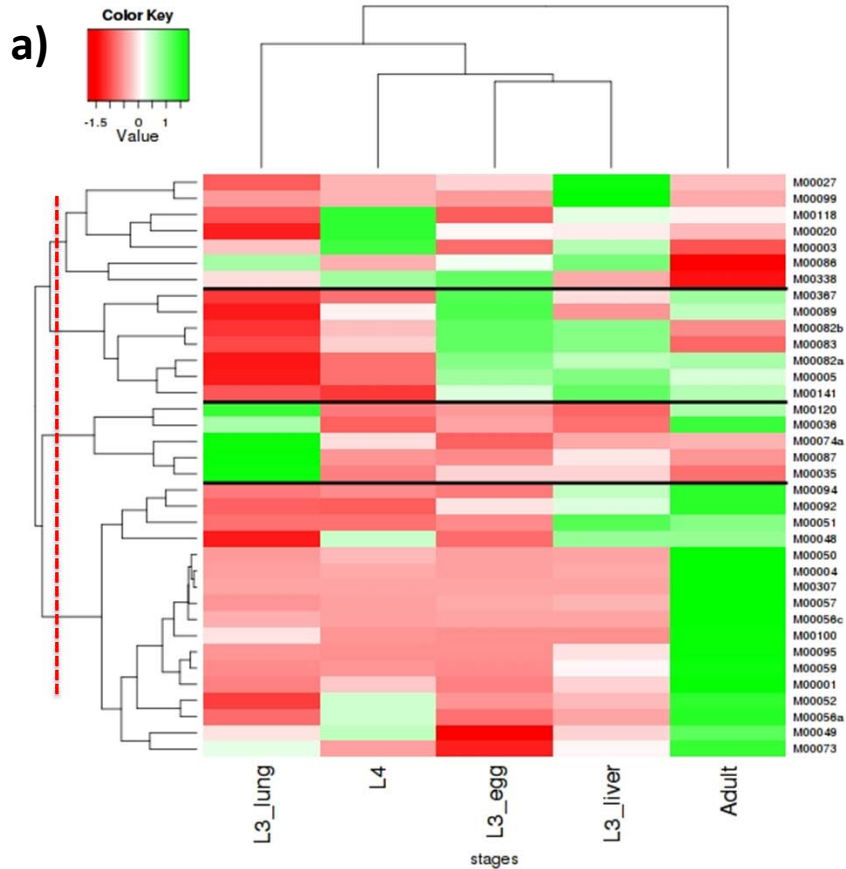

**b)**

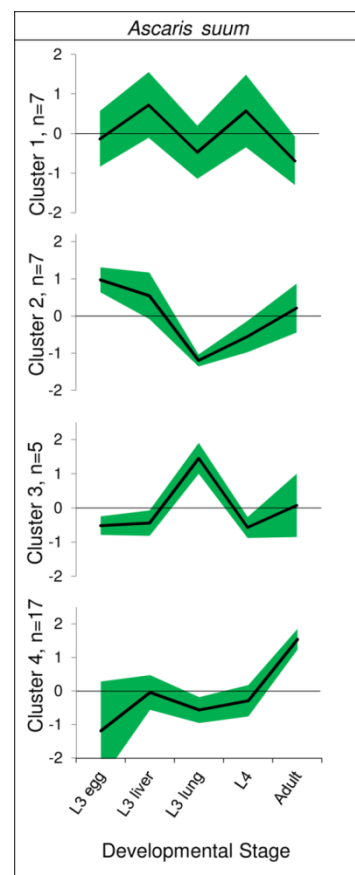

**c)**

*N. americanus* abundance ratio

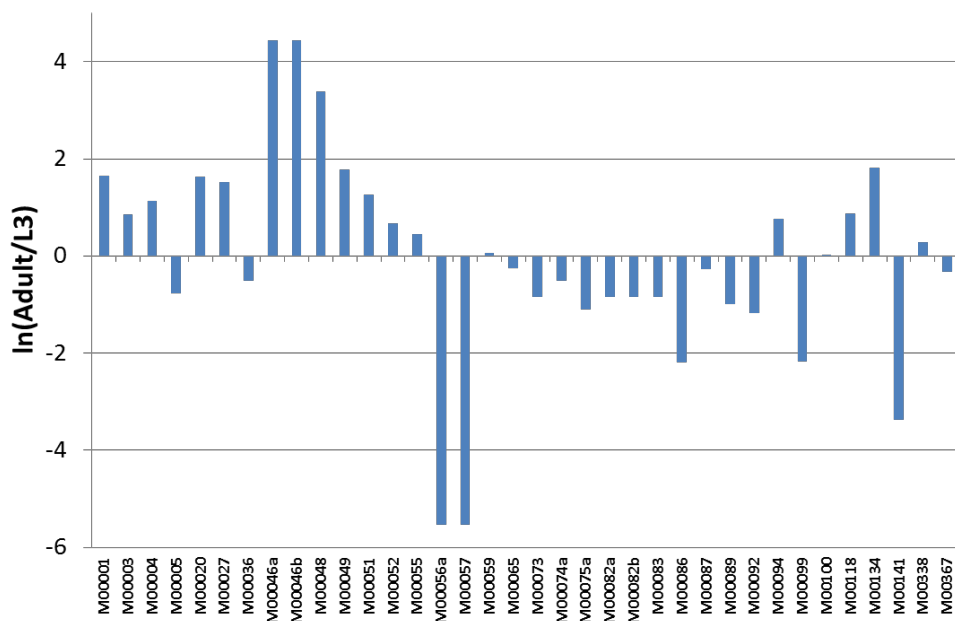

Supplement: S13 Fig — a) A Heatmap showing clustering of modules and developmental stages based on module abundance patterns in A. suum. The adult stage abundances have been combined here to aid in obtaining clusters more relevant to developmental differences. There are clear differences in stages here, with some modules very clearly showing high overabundance in only 1 of the developmental stages (especially L3_lung and Adult). The red line divides modules into 4 clusters that share abundance profiles in these developmental stages. The heatmap rows of these clusters are separated by a black bar for clarity. b) Developmental stage based metabolic profiles A. suum. The cluster characteristics are discussed in S1 Text. These plots are analogous to those presented in Fig 4 for C. elegans and B. malayi. c) Module abundance ratios (in log2 units) for Necator americanus metabolic modules. Modules 56a and 57 have 0 Adult/L3 abundance ratio, and there bars on the graph are indicative of-inf. 17 out of 36 modules have a significant difference between L3 and adult abundances (|ln(Adult/L3)|>1), with 10 modules being overabundant in Adult stage and 7 in the infective L3 stage. (PDF) [file pntd.0003788.s013.pdf]

a)

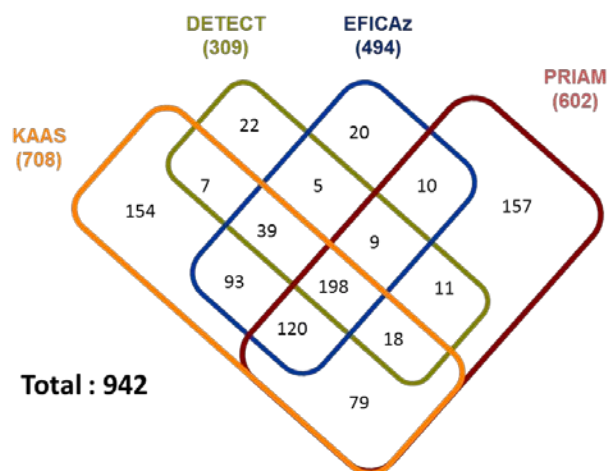

b)

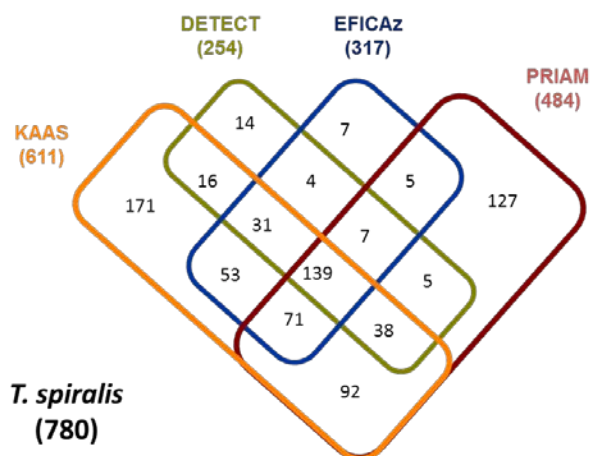

c)

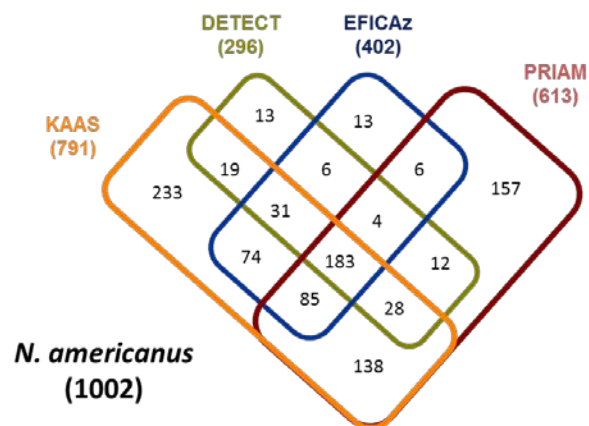

d)

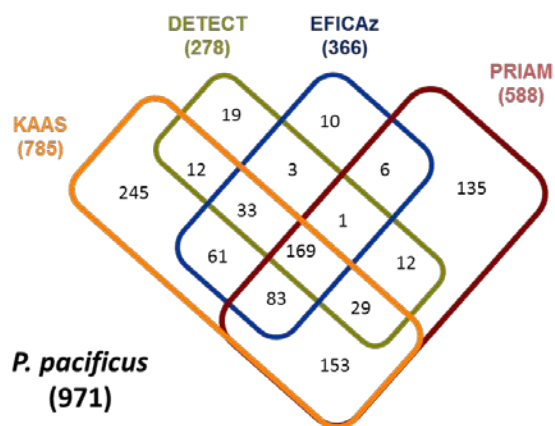

Supplement: S14 Fig — a) Using the 3 methods expands the annotated enzyme set by an average of 33.1% w.r.t. annotations based only on KAAS in C. elegans. Allowing only those additional annotations that are supported by at least 2 methods expands our annotation set for C. elegans by 4.9%. (b-d) Including 3 parasitic nematodes (b: T. spiralis, c: N. americanus, d: P. pacificus) resulted in the mean expansion of their enzyme activity detection by 27.8%. Allowing only new high confidence annotation (as determined by their confirmation by at least 2 of the methods) expands our enzyme annotation by 3.7%. (PDF) [file pntd.0003788.s014.pdf]
