# Supplementary material for: Pan-phylum Comparison of Nematode Metabolic Potential
Source: PLoS Negl Trop Dis. 2015 May 22;9(5):e0003788. doi: 10.1371/journal.pntd.0003788 (PMC4441503; doi:10.1371/journal.pntd.0003788)
Supplement: S5 Fig — This is a version of Fig 2B, with complete module names replacing KEGG module IDs. (PDF) [file pntd.0003788.s005.pdf]

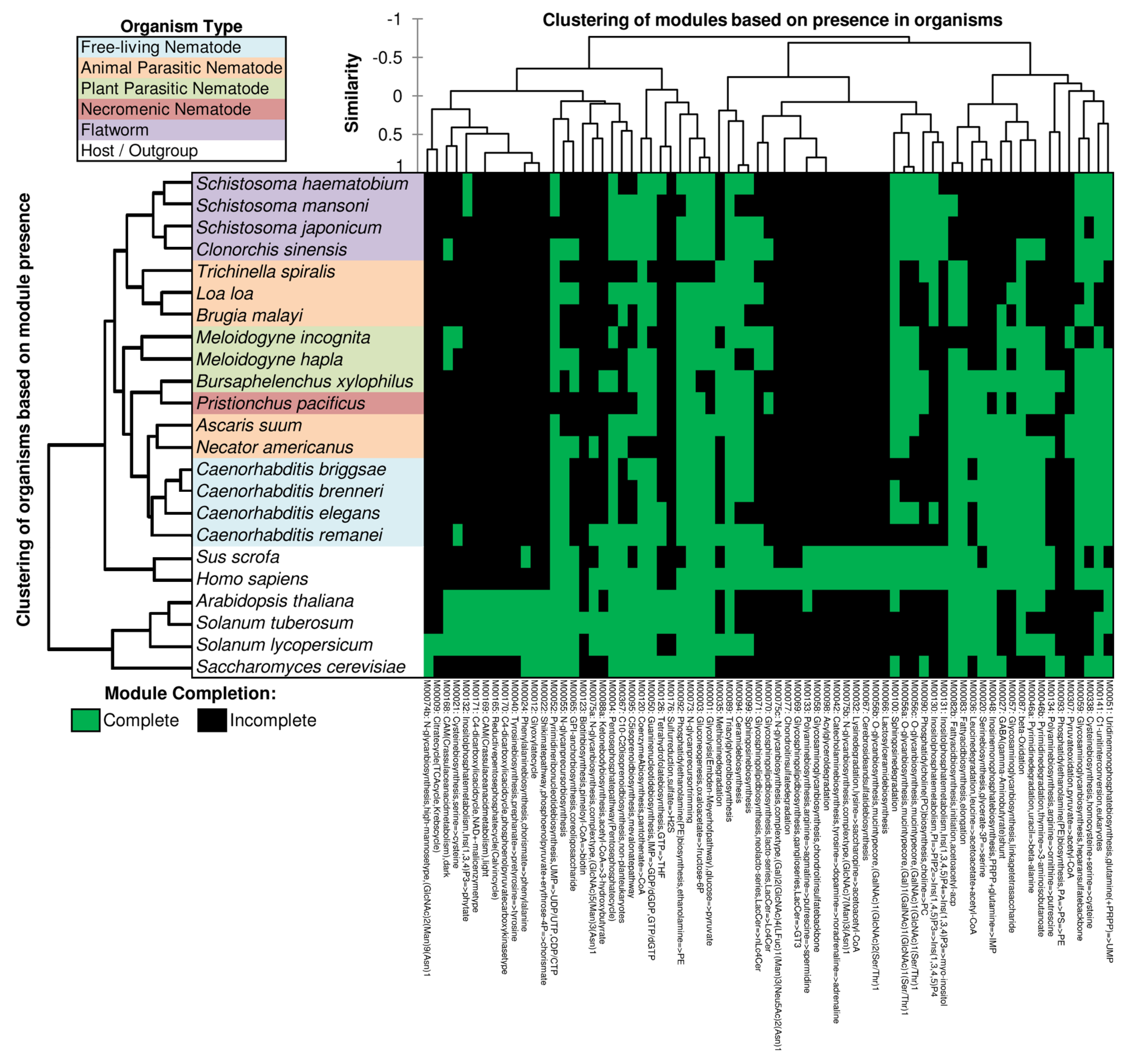

Organism Type
Free-living Nematode
Animal Parasitic Nematode
Plant Parasitic Nematode
Necromenic Nematode
Flatworm
Host / Outgroup
Clustering of modules based on presence in organisms
Similarity
-1
-0.5
0
0.5
1
Clustering of organisms based on module presence
Schistosoma haematobium
Schistosoma mansoni
Schistosoma japonicum
Clonorchis sinensis
Trichinella spiralis
Loa loa
Brugia malayi
Meloidogyne incognita
Meloidogyne hapla
Bursaphelenchus xylophilus
Pristionchus pacificus
Ascaris suum
Necator americanus
Caenorhabditis briggsae
Caenorhabditis brenneri
Caenorhabditis elegans
Caenorhabditis remanei
Sus scrofa
Homo sapiens
Arabidopsis thaliana
Solanum tuberosum
Solanum lycopersicum
Saccharomyces cerevisiae
Module Completion:
Complete
Incomplete
M00074b: N-glycanbiosynthesis,high-mannosetype,(GlcNAc)2(Man)9(Asn)1
M00009: Citratecycle(TCAcycle,Krebscycle)
M00168: CAM(Crassulaceanacidmetabolism),dark
M00021: Cysteinebiosynthesis,serine=>cysteine
M00132: Inositolphosphatemetabolism,Ins(1,3,4)P3=>phytate
M00171: C4-dicarboxylicacidcycle,NAD+-malicenzymetype
M00169: CAM(Crassulaceanacidmetabolism),light
M00165: Reductivepentosephosphatecycle(Calvincycle)
M00170: C4-dicarboxylicacidcycle,phosphoenolpyruvatecarboxykinasetype
M00040: Tyrosinebiosynthesis,prephanate=>pretyrosine=>tyrosine
M00024: Phenylalaninebiosynthesis,chorismate=>phenylalanine
M00012: Glyoxylatecycle
M00022: Shikimatepathway,phosphoenolpyruvate+erythrose-4P=>chorismate
M00052: Pyrimidineribonucleotidebiosynthesis,UMP=>UDP/UTP,CDP/CTP
M00055: N-glycanprecursorbiosynthesis
M00065: GPI-anchorbiosynthesis,coreoligosaccharide
M00123: Biotinbiosynthesis,pimeloyl-CoA=>biotin
M00075a: N-glycanbiosynthesis,complextype,(GlcNAc)5(Man)3(Asn)1
M00088a: Ketonebodybiosynthesis,acetyl-CoA=>3-hydroxybutyrate
M00004: Pentosephosphatepathway(Pentosephosphatecycle)
M00367: C10-C20isoprenoidbiosynthesis,non-planteukaryotes
M00095: C5isoprenoidbiosynthesis,mevalonatepathway
M00120: CoenzymeAbiosynthesis,pantothenate=>CoA
M00050: Guaninenucleotidebiosynthesis,IMP=>GDP/dGDP,GTP/dGTP
M00126: Tetrahydrofolatebiosynthesis,GTP=>THF
M00176: Sulfurreduction,sulfate=>H2S
M00092: Phosphatidylethanolamine(PE)biosynthesis,ethanolamine=>PE
M00073: N-glycanprecursortrimming
M00003: Gluconeogenesis,oxaloacetate=>fructose-6P
M00001: Glycolysis(Embden-Meyerhofpathway),glucose=>pyruvate
M00035: Methioninedegradation
M00089: Triacylglycerolbiosynthesis
M00094: Ceramidebiosynthesis
M00099: Sphingosinebiosynthesis
M00071: Glycosphingolipidbiosynthesis,neolacto-series,LacCer=>nLc4Cer
M00075c: N-glycanbiosynthesis,complextype,(Gal)2(GlcNAc)4(LFuc)1(Man)3(Neu5Ac)2(Asn)1
M00070: Glycosphingolipidbiosynthesis,lacto-series,LacCer=>Lc4Cer
M00077: Chondroitinsulfatedegradation
M00069: Glycosphingolipidbiosynthesis,ganglioseries,LacCer=>GT3
M00133: Polyaminebiosynthesis,arginine=>agmatine=>putrescine=>spermidine
M00058: Glycosaminoglycanbiosynthesis,chondroitinsulfatebackbone
M00098: Acylglyceroldegradation
M00042: Catecholaminebiosynthesis,tyrosine=>dopamine=>noradrenaline=>adrenaline
M00075b: N-glycanbiosynthesis,complextype,(GlcNAc)7(Man)3(Asn)1
M00032: Lysinedegradation,lysine=>saccharopine=>acetoacetyl-CoA
M00056b: O-glycanbiosynthesis,mucintypecore,(GalNAc)1(GlcNAc)2(Ser/Thr)1
M00067: Cerebrosidesulfatidebiosynthesis
M00066: Lactosylceramidebiosynthesis
M00100: Sphingosinedegradation
M00056c: O-glycanbiosynthesis,mucintypecore,(GalNAc)1(GlcNAc)1(Ser/Thr)1
M00056a: O-glycanbiosynthesis,mucintypecore,(Gal)1(GalNAc)1(GlcNAc)1(Ser/Thr)1
M00090: Phosphatidylcholine(PC)biosynthesis,choline=>PC
M00130: Inositolphosphatemetabolism,PI=>PIP2=>Ins(1,4,5)P3=>Ins(1,3,4,5)P4
M00131: Inositolphosphatemetabolism,Ins(1,3,4,5)P4=>Ins(1,3,4)P3=>myo-inositol
M00082b: Fattyacidbiosynthesis,initiation,acetoacetyl-acp
M00083: Fattyacidbiosynthesis,elongation
M00036: Leucinedegradation,leucine=>acetoacetate+acetyl-CoA
M00020: Serinebiosynthesis,glycerate-3P=>serine
M00048: Inosinemonophosphatebiosynthesis,PRPP+glutamine=>IMP
M00027: GABA(gamma-Aminobutyrate)shunt
M00057: Glycosaminoglycanbiosynthesis,linkagetetrasaccharide
M00087: beta-Oxidation
M00046a: Pyrimidinedegradation,uracil=>beta-alanine
M00046b: Pyrimidinedegradation,thymine=>3-aminoisobutanoate
M00134: Polyaminebiosynthesis,arginine=>ornithine=>putrescine
M00093: Phosphatidylethanolamine(PE)biosynthesis,PA=>PS=>PE
M00307: Pyruvateoxidation,pyruvate=>acetyl-CoA
M00059: Glycosaminoglycanbiosynthesis,heparansulfatebackbone
M00338: Cysteinebiosynthesis,homocysteine+serine=>cysteine
M00141: C1-unitinterconversion,eukaryotes
M00051: Uridinemonophosphatebiosynthesis,glutamine(+PRPP)=>UMP
