# Supplementary material for: Pan-phylum Comparison of Nematode Metabolic Potential
Source: PLoS Negl Trop Dis. 2015 May 22;9(5):e0003788. doi: 10.1371/journal.pntd.0003788 (PMC4441503; doi:10.1371/journal.pntd.0003788)
Supplement: S10 Fig — a. N-glycan variants among helminths are represented as single elaborated structures—in living organisms, N-glycan structures are heterogeneous. Glycosyltransferases create bonds between monosaccharides, given as configuration (alpha α or beta β) along with linkage position (2,3,4,6). Our metabolic module analysis is consistent with the backbone structure of N-glycans (chitobiose trimannose). Consistent with previous work, nematoda and platyhelminths under study lack sialyltransferases found in mammals. Previous biochemical analysis of helminth N-glycans has demonstrated phosphorylcholine and xylose modifications not found in humans (boxed). Immunologically-important glycosylation patterns for parasitic worms are: Lewis X (Galβ1–4[Fucα1–3]GlcNAc-) and LDNF (GalNAcβ1–4[Fucα1–3]GlcNAc-), as described in the text. b. O-glycans of helminths. Mammalian O-glycans are frequently sialylated (α2,3 or α2,6). Elaborated mammalian glycans built on Core 2 and Core 4 are depicted here, but other sialylated structures are also synthesized. Carbohydrates lacking sialic acid, including T antigen and Tn antigen (right) have been detected experimentally in multiple helminths. In contrast to human, O-glycans of nematodes and flatworms can be glucuronidated and methylated—these features were not explicitly detected by computational analysis using KEGG modules. (PDF) [file pntd.0003788.s010.pdf]

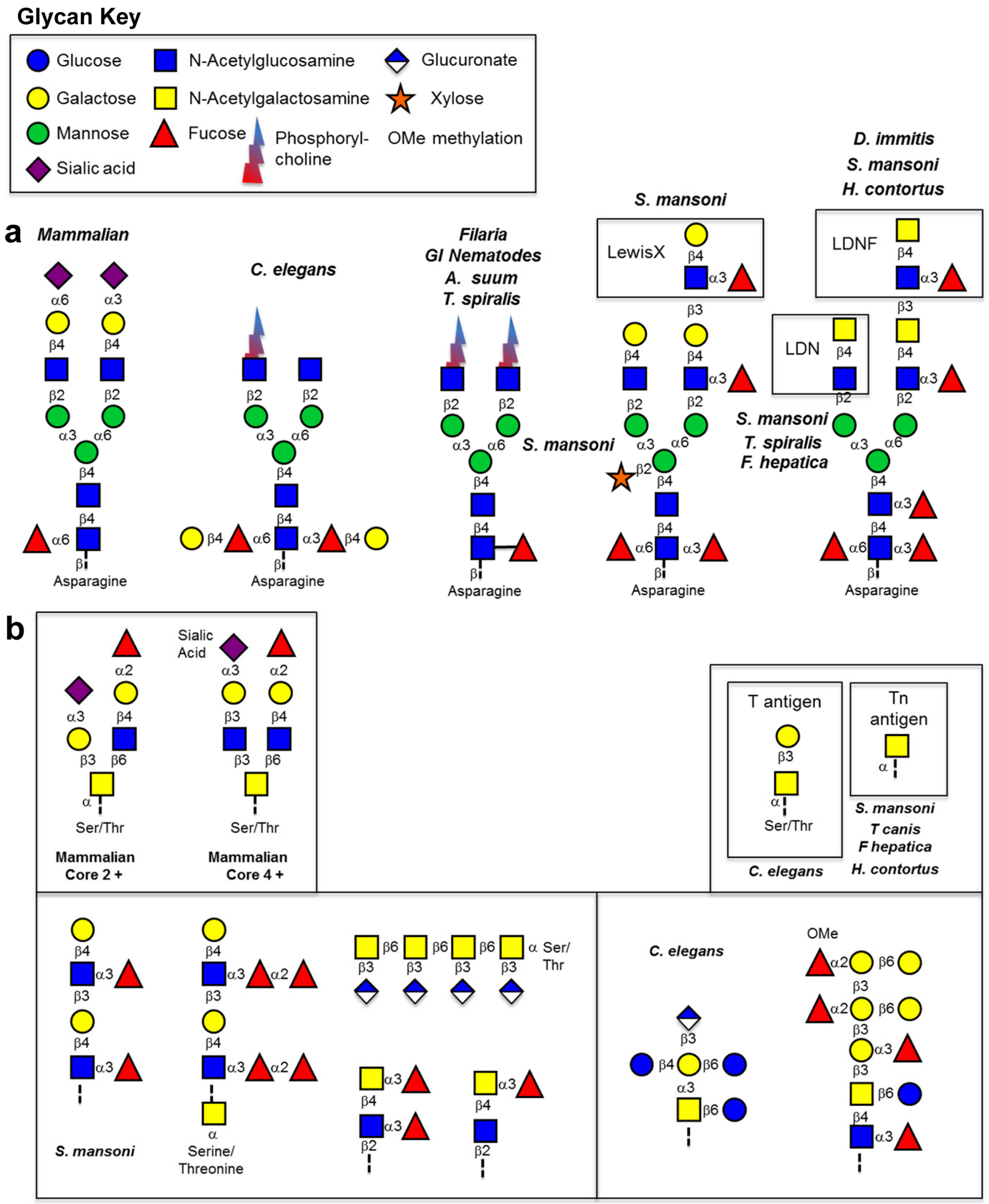
Glycan Key
Glucose
N-Acetylglucosamine
Glucuronate
Galactose
N-Acetylgalactosamine
Xylose
Mannose
Fucose
Phosphoryl-
choline
OMe methylation
Sialic acid
a
Mammalian
C. elegans
Filaria
GI Nematodes
A. suum
T. spiralis
S. mansoni
LewisX
D. immitis
S. mansoni
H. contortus
LDNF
LDN
S. mansoni
T. spiralis
F. hepatica
Asparagine
b
Sialic
Acid
Ser/Thr
Mammalian
Core 2 +
Mammalian
Core 4 +
T antigen
Tn
antigen
C. elegans
S. mansoni
T canis
F hepatica
H. contortus
S. mansoni
Serine/
Threonine
Ser/
Thr
C. elegans
OMe
