# Supplementary material for: Pan-phylum Comparison of Nematode Metabolic Potential
Source: PLoS Negl Trop Dis. 2015 May 22;9(5):e0003788. doi: 10.1371/journal.pntd.0003788 (PMC4441503; doi:10.1371/journal.pntd.0003788)
Supplement: S1 Text — (DOCX) [file pntd.0003788.s023.docx]

# S1 Text Metabolic dynamics in parasitic worms

*Ascaris suum -* We analyzed data for 4 pre-adulthood developmental stages (L3 egg, L3 liver, L3 lung and L4) and 4 adult stage tissues (male/female muscle and reproductive tissue). In order to obtain a development stage-relevant clustering, we combined the 4 adult tissues by using their mean abundance as the representative abundance for adult stage (S13a Fig). One noticeable characteristic differentiating this from *B. malayi* and, especially, *C. elegans* is that a vast majority of modules show very high over or underabundance only in 1 or 2 stages, suggesting a higher level of developmental stage specific metabolism in *A. suum* as compared to the other 2 organisms. This is especially true for clusters 3 and 4 that have significant overabundance only in L3 lung stage and the adult stage respectively (S13b Fig). Cluster 3 comprises of modules for beta oxidation, degradation of leucine and methionine, and CoA and N-glycan biosynthesis. Cluster 4 includes modules for biosynthesis of Glycolysis, pentose phosphate pathway, N-glycan precursor trimming, sphingosine degradation, pyruvate oxidation and biosynthesis of C5 isoprenoids, Ceramide, PE, O-glycans, glycosaminoglycans, Adenine and Guanine nucleotides and pyrimidine ribonucleotides, and monophosphates of Inosine and Uridine. Cluster 2 is distinguished by uniquely high abundance in the L3 egg stage and underabundance in the L3 lung stage. The modules in this cluster are related to biosynthesis of fatty acids, triacylglycerol, PRPP and C10-C20 isoprenoids. Cluster 1 shows relatively uniform abundance throughout the L3 to Adult stages, with moderate increases in L3 liver and L4 stages. This cluster comprises of modules for gluconeogenesis, GABA shunt, and biosynthesis of Serine, Cysteine, Sphingosine, Glutathione and acyl-CoA.

## *Necator americanus -* Since we only had expression data for 2 developmental stages of *N. americanus*, we decided to find modules relevant to development by looking at abundance ratios between the two stages. The ratio of abundances in Adult and L3 stages was used as the metric to determine over or underabundance. ln(Adult/L3) was plotted (S13c Fig), and values higher than 1 (Adult overabundant) or less than 1 (L3 overabundant) were used as thresholds for significant differential abundance.

One of the 37 modules potentially complete in the hookworm had no detectable expression in both these stages (M00307, Pyruvate oxidation, pyruvate => acetyl-CoA). The abundance ratios of the other 36 modules are shown in natural log units in S13c Fig. 17 out of these 36 have a ln(abundance ratio) of more than 1 (i.e. a higher than 2.7-fold difference in abundances in the two stages), with 10 modules being more abundant in the parasitic adult stage, and 7 modules more abundant in the infective L3 stage. The modules that are overabundant in the infective L3 stage in hookworm are primarily related to glycan and lipid synthesis and metabolism. The parasitic blood feeding adult stage has overabundance of modules for glycolysis and synthesis and metabolism of amino acids and nucleotides.
